# Core genes of biomineralization and *cis*-regulatory long non-coding RNA regulate shell growth in bivalves

**DOI:** 10.1016/j.jare.2023.11.024

**Published:** 2023-11-22

**Authors:** Maoxiao Peng, João C.R. Cardoso, Gareth Pearson, Adelino VM Canário, Deborah M. Power

**Affiliations:** a Comparative Endocrinology and Integrative Biology, Centre of Marine Sciences, Universidade do Algarve, Campus de Gambelas, 8005-139 Faro, Portugal; b Biogeographical Ecology and Evolution, Centre of Marine Sciences, Universidade do Algarve, Campus de Gambelas, 8005-139 Faro, Portugal; c International Research Center for Marine Biosciences, Ministry of Science and Technology, Shanghai Ocean University, Shanghai, China; d Key Laboratory of Exploration and Utilization of Aquatic Genetic Resources, Ministry of Education, Shanghai Ocean University, Shanghai, China

**Keywords:** Bivalve, Mantle transcriptomes, LncRNA *cis*-regulation, Shell asymmetry, Shell biomineralization toolbox

## Abstract

**Introduction:**

Bivalve molluscs are abundant in marine and freshwater systems and contribute essential ecosystem services. They are characterized by an exuberant diversity of biomineralized shells and typically have two symmetric valves (a.k.a shells), but oysters (Ostreidae), some clams (Anomiidae and Chamidae) and scallops (Pectinida) have two asymmetrical valves. Predicting and modelling the likely consequences of ocean acidification on bivalve survival, biodiversity and aquaculture makes understanding shell biomineralization and its regulation a priority.

**Objectives:**

This study aimed to a) exploit the atypical asymmetric shell growth of some bivalves and through comparative analysis of the genome and transcriptome pinpoint candidate biomineralization-related genes and regulatory long non-coding RNAs (LncRNAs) and b) demonstrate their roles in regulating shell biomineralization/growth.

**Methods:**

Meta-analysis of genomes, *de novo* generated mantle transcriptomes or transcriptomes and proteomes from public databases for six asymmetric to symmetric bivalve species was used to identify biomineralization-related genes. Bioinformatics filtering uncovered genes and regulatory modules characteristic of bivalves with asymmetric shells and identified candidate biomineralization-related genes and lncRNAs with a biased expression in asymmetric valves. A shell regrowth model in oyster and gene silencing experiments, were used to characterize candidate gene function.

**Results:**

Shell matrix genes with asymmetric expression in the mantle of the two valves were identified and unique *cis*-regulatory lncRNA modules characterized in Ostreidae. LncRNAs that regulate the expression of the tissue inhibitor of metalloproteinases gene family (*TIMPDR)* and of the shell matrix protein domain family (*SMPDR*) were identified. *In vitro* and *in vivo* silencing experiments revealed the candidate genes and lncRNA were associated with divergent shell growth rates and modified the microstructure of calcium carbonate (CaCO_3_) crystals.

**Conclusion:**

LncRNAs are putative regulatory factors of the bivalve biomineralization toolbox. In the Ostreidae family of bivalves biomineralization-related genes are *cis*-regulated by lncRNA and modify the planar growth rate and spatial orientation of crystals in the shell.

## Highlights

• In bivalves with asymmetric shells, DEGs are more abundant in the flat valve mantle.
• LncRNA are new members of the bivalve biomineralization toolbox.
• Biomineralization-related genes in the mantle are under *cis* regulation by lnRNAs.
• Asymmetric Ostreidae family members share conserved regulatory gene modules.
• Gene modules regulate calcium carbonate crystal growth and spatial orientation.

## Graphical abstract

**Figure:**
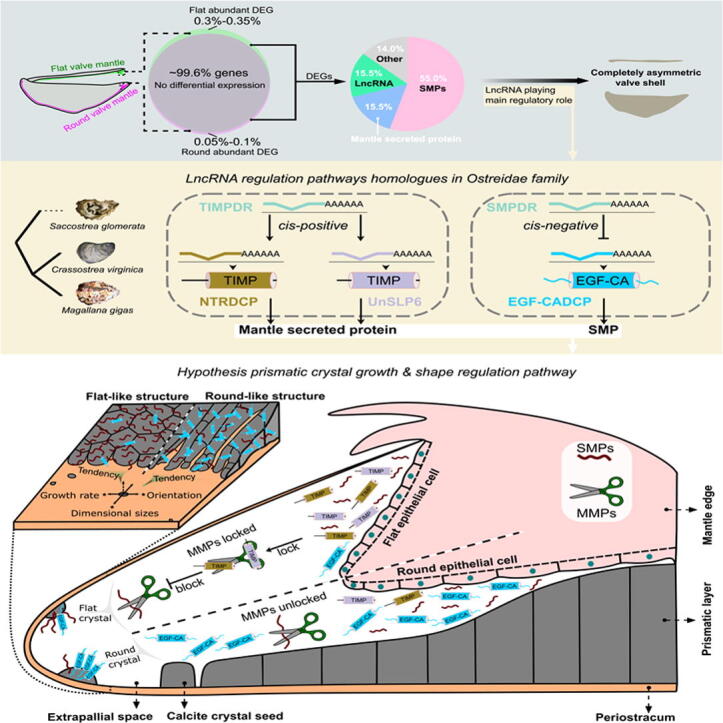

## Introduction

A renewed interest in mollusc shells and how they are produced has arisen because of current ocean acidification trends and its implication for shell calcification and Mollusca survival, biodiversity and the burgeoning aquaculture industry [1], [2], [3]. The biomineralized shell of the speciose molluscs contributed to their evolutionary success during and after the Cambrian explosion [4], [5]. Shell microstructure and architecture has been characterised in a very small proportion of all bivalves but reveals they are extraordinarily diverse [6] and result from crystallization of calcium carbonate (CaCO_3_) in a soft organic matrix made up of proteins, acidic polysaccharides and chitin [7]. The shell gland in mollusc embryos and the outer mantle epithelium and haemocytes (responsible for calcium and carbonate ion transport [8], [9], [10][[86], [87]]) in the extrapallial fluid in subsequent stages produce the shell [11]. The mantle has been extensively studied as the main shell biomineralizing tissue and although the shell organic matrix accounts for less than 5 % of its composition, shell diversity has been assigned to the rapidly evolving mantle secretome [7], [12], [13].

Asymmetrical shell growth has been mostly studied in the gastropods, the most specious class of molluscs, where most species have inherited one helical asymmetrical coiled shell [14], [15], [16], [17], [18]. Although their soft body in common with other bilaterian organisms exhibits external left/right (LR) symmetry along the sagittal plane [19]. Specialization of the shell-secreting mantle epithelium in gastropods controls shell morphology and structure. In the basket whelk (*Tritia obsoleta*) and ram's horn snail (*Planorbella sp*.) specialization of the mantle shell margin occurs during larval development and in *Tritia* is associated with regional expression of regulatory genes [20]. In the pond snail (*Lymnaea stagnalis*) asymmetric gene expression of shell matrix proteins (SMPs) by the mantle contribute to shell asymmetry [18], [20]. Overall, asymmetric shell growth in gastropods is a consequence of body torsion and differential gene expression during development, however in other Mollusca, such as the bivalves, this process remains unclear.

Bivalve molluscs have a shell composed of two symmetric valves, although a few modern bivalves such as the oysters (Ostreidae), clams (Anomiida and Chamidae) and some scallops (Pectinida) have asymmetric valves indicating that shell asymmetry emerged at least twice during the bivalve radiation [21]. Why oyster and scallop valves deviate from the common symmetric pattern of bivalves is an evolutionary enigma [22]. In most oysters, the right valve is flat, and the left valve attached to the substrate is convex (cupped or round) [23]. The explanation for bivalve shell diversity is thought to lie in the recently characterized, highly evolved and diverse bivalve “biomineralization toolbox” [13], [24], [25], [26], which contains ion transporters, SMPs and enzymes [9], [27], [28]. But if there are master regulator genes is a mystery and it has been hypothesised that shell microstructure and diversity of shell shapes arose from a conserved upstream *cis*-regulatory network acting on the biomineralization toolbox genes in the bivalve mantle [25], [29]. *Nacrein* and *SLC2A1* (Solute Carrier Family 2 Member 1) toolbox genes were proposed as candidates for shell asymmetry in the Hong Kong oyster *Magallana hongkongensis* (previously called *Crassostrea hongkongensis*) [30]. In contrast, in the adult Pacific oysters (*M. gigas*), factors regulating body plan asymmetry in vertebrates such as the Nodal cascade [31], [32] were associated with valve asymmetry [33].

Long non-coding RNAs (lncRNAs) are a group on non-coding RNAs (ncRNAs) that are generally defined as transcripts of more than 200 nucleotides that are not translated into protein [34] and play an important multilevel regulatory role in various biological processes including development, cell differentiation, immune responses and tissue regeneration [35], [36], [37], [38], [39], [40], [41], [42], [43]. To date only a few lncRNAs have been functionally characterized in Mollusca [44]. In the bivalve, *Pinctada fucata*, lncRNAs (lncMSEN1 and lncMSEN2) were reported to be associated with the response to poly I:C and LPS stimulation and with maintaining shell nacreous layer microstructure [38], [39], [40]. In addition, lncIRF-2 was reported to play a role in the immune response [37]. Protein-lncRNA interactions are reported to regulate pigment synthesis, shell colour and larval development in *M. gigas* [35], [36] and although lncRNAs, are differentially expressed in the mantle of the cupped (round) and flat valves, their possible roles in shell shape and shell structure have not been considered [30], [33].

Following Krogh’s principle [45], we selected bivalves with completely asymmetric shells and bivalves with slightly asymmetric shells or totally symmetric shells as models and took a comparative approach to identify coding and non-coding RNA associated with the regulation of shell matrix crystallization and growth. We reasoned that asymmetric shells arise from different rates of growth, but that the similar chemical composition of the valves means that the basic set of shell building genes in both mantles should be similar, and that by identifying differentially expressed genes in symmetric and asymmetric bivalves it should be possible to tease out the most important gene transcripts and regulatory factors for the trait. This was done using a step wise analysis of molecular resources from six symmetric to asymmetric bivalves to identify a) similarities and differences between mantle transcriptomes, b) core biomineralization-related genes and protein motifs in the mantle, c) biomineralization toolbox genes with biased expression in asymmetry and d) lncRNA and their putative binding partners. Using *meta*-analysis of available data, we identified collinear *cis*-regulatory regions and putative binding pairs composed of lncRNA and biomineralization toolbox genes in the evolutionary proximate asymmetric species *M. gigas*, *C. virginica* and *S. glomerata*. Targeted knock-down in *M. gigas* of the identified lncRNA *in vitro* down-regulated the expression of candidate biomineralization genes. Down-regulation of the genes by knock-down *in vivo* led to divergent rates of shell deposition and modified crystal growth. Overall, we conclude that valve asymmetry in *M. gigas* most likely results from *cis*-regulation by lncRNAs of specific biomineralization toolbox genes leading to their biased expression in the mantle, and reduced rates of biomineralization in the flat valve.

## Materials and methods

### Ethics statement

All experiments involving animals were conducted according to the ethical policies and procedures approved by the ethics committee of the Centre of Marine Sciences, University of Algarve, Portugal (Approval no. ICNF_327/2022/CAPT).

### Animals, sampling, and experimental conditions

Adults from aquaculture production (length 9.2 ± 0.7 cm, width 5.0 ± 0.5 cm, ∼1.5-years-old) and juvenile (length 2.5 ± 0.3 cm. width 1.8 ± 0.2 cm, ∼ 5-month-old) *M. gigas* were kindly donated by Francois Hubert from the Bivalvia company located in the Ria Formosa, Olhão (Portugal). Wild adult (length 6.3 ± 0.1 cm, width 3.3 ± 0.2 cm, ∼6-month-old) *Mytilus galloprovincialis* were captured by hand from the Ria Formosa (Faro, 37°00′32″N, 7°59′40″W). Animals were transported live to CCMAR experimental facilities and were acclimated for 3 days in 5 L tanks (25 (length) × 17 (width) × 15 (height), cm) filled with aerated seawater (37 ppt) collected from their natural environment before experimentation. All tanks were maintained at room temperature (20–––23 °C) and animals were fed once a day (0.002 g / g body weight) with a commercial mixture of dried microalgae (Phytobloom, Reef Feed, Portugal, Olhão). All experiments were run under similar conditions. Before tissue collection animals were anaesthetized with iced seawater and the mantles from both valves (right/flat and left/round or convex valve) were collected. The mantle margin from juvenile *M. gigas* was collected for transcriptome generation, expression analysis, and to assess the impact of *in vivo* shell damage-repair assays on target gene expression. The mantle margin from adult *M. galloprovincialis* was collected for transcriptome generation. The mantle margin from adult *M. gigas* was also collected for *ex vivo* siRNA experiments and for analysis of the subcellular localization of lncRNA. For the transcriptomics analysis, the mantle edge of animals was immediately sampled after collection, snap-frozen in liquid nitrogen and stored at −80 °C until RNA extractions. For details regarding RNA extractions, cDNA synthesis and small-scale gene expression analyses refer to SI Appendix.

### Multi-omics analysis of bivalve mantle transcriptomes

The mantles from right/flat and left/round valves of juvenile *M. gigas* (*n* = 6/shell side) and adult *M. galloprovincialis* (*n* = 6/shell side) were collected and RNA extracted. Library preparation and sequencing of *M. gigas* (*n* = 3, pools of RNA from 2 individuals) was performed at Shanghai Ocean University Sequencing Service (Shanghai, China) and index codes were used to attribute sequences to individual samples (Supplementary Table 1). Library preparation and sequencing of *M. galloprovincialis* (*n* = 3, pools of RNA from 2 individuals) was performed at Novogene Europe Co., Ltd Sequencing Service and index codes were used to attribute sequences to individual samples (Supplementary Table 1). For details regarding protocols of library preparation (for *M. gigas* and *M. galloprovincialis*), transcriptome quality control, assembly, and the calculation of differentially expressed genes, see SI Appendix. To increase the robustness and data for the mantle transcriptome analysis publicly available mantle transcriptomes (raw data) from two other bivalves, the asymmetric shelled *Mizuhopecten yessoensis* [46] and the slightly asymmetric shelled *P. fucata* [30] was obtained from NCBI (Supplementary Table 1). These two species were selected because of the public availability of sequencing data from libraries for flat and round valve mantles and the availability of reference genomes (*M. yessoensis* NCBI Accession: GCA_002113885.2 [47] and *P. fucata*, ver3.0 [48], respectively) for mapping of reads using the same methods described for *M. gigas* and *M. galloprovincialis* (see SI Appendix). In this study, the flat valve, of the asymmetric bivalve, was assigned to the right valve of slightly asymmetric and symmetric bivalves, and the round (or cupped) valve (asymmetric bivalves) was assigned to the left valve (slightly asymmetric and symmetric bivalve).

### Identification of biomineralization toolbox genes and regulatory factors in mantle

DE coding (DEc) and DE non-coding (DEnc) transcripts were annotated. DEc were also annotated using blastp against NCBI (nr) and Swiss-Prot databases, respectively. DEc in *M. gigas*, *M. yessoensis*, *P. fucata* and *M. galloprovincialis* were annotated for subcellular localization using three programmes, for details see SI Appendix. Protein domains were identified using Pfam (ver 34.0) (https://pfam.xfam.org/) and compared with the *M. gigas* shell proteome [9] to identify common domains. Protein motifs were identified using ProminTools [49] (Supplementary Fig 1), for details see SI Appendix. The *M. gigas* shell proteome was compared to secreted DEc of *M. yessoensis*, *P. fucata* and *M. galloprovincialis* to identify common asymmetric-related protein domains/motifs. DE lncRNAs (DEnc) were identified based on *M. gigas* and *M. galloprovincialis* genome annotations, respectively. Their full-length nucleic acid sequence and authenticity as non-protein coding gene transcripts was reconfirmed, for details see the SI Appendix. Confirmed DEnc of *M. gigas* were assigned an arbitrary name lncRNA1-20, and lncRNA 3 and lncRNA 20 and after functional validation were named *TIMPDR* and *SMPDR*, respectively. Publicly available bivalve transcriptome data from several tissues, larva at different developmental stages and shell damage-repair experiments of *M. gigas* [50] was analysed and lncRNA candidates identified. Publicly available transcriptomes were downloaded from the SRA database of NCBI (Supplementary Table 1) and expression levels of candidate lncRNA were calculated in FPKM. DEnc that were (a) abundant in the mantle and (b) responded to shell-damage repair were considered putative candidates for shell formation. LncRNAs that were highly expressed in pediveliger larvae (before metamorphosis), spat and juvenile stages (after metamorphosis) were selected as candidates for shell shape transition/formation (from symmetry to asymmetry).

**Fig. 1:**
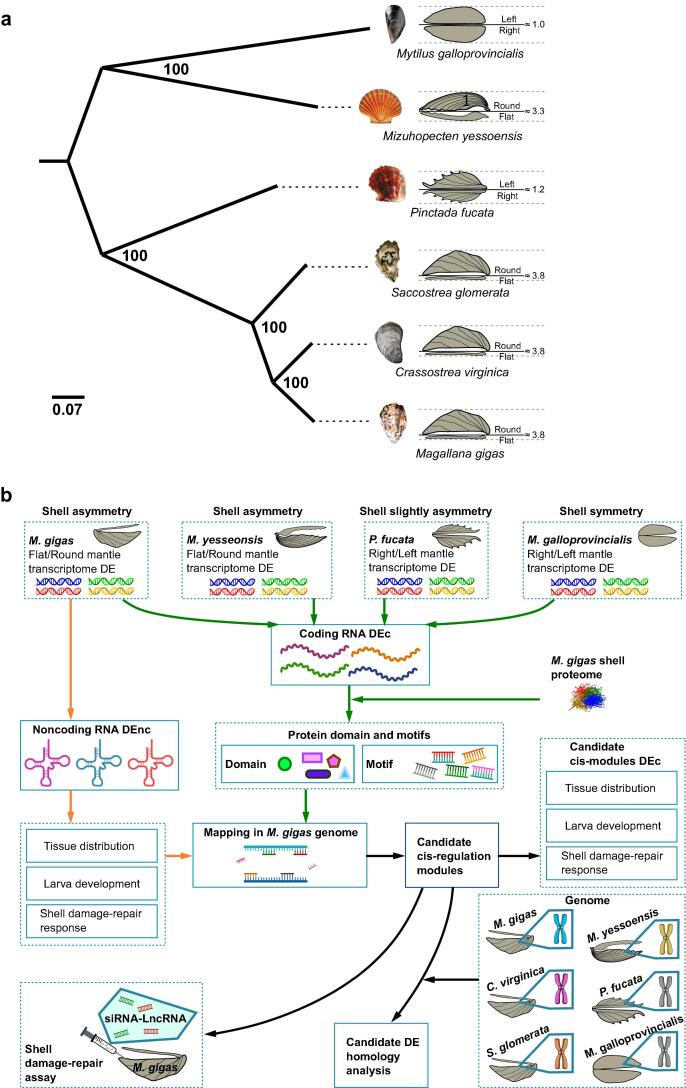
**Workflow of the strategy to screen DEGs that regulate shell asymmetry of the bivalves in this study.** (**a**) Phylogenetic relationship of the bivalves studied. The phylogenetic tree was constructed using the Orthofinder (ver 2.5.4, methods see SI Appendix) and the ML method. The shell height ratio and shell pattern plots are shown on the right side of the phylogenetic tree. Species with asymmetric shells (*M. gigas*, *C. virginica*, *S. glomerata*, *M. yessoensis*), slightly asymmetric shell (*P. fucata*), and symmetric shell (*M. galloprovincialis*) are included in this study. Shell height ratio of *M. gigas* and *M. galloprovincialis* were measured in experimental specimens (*n* = 30), *M. yessoensis* shell height ratio data was provided by Xueshu Zhang, Dalian Ocean University (*n* = 30), *P. fucata* shell height ratio data was provided by Junlong Sun, Hainan University (*n* = 30), *C. virginica* and *S. glomerata* shell height ratio were measured using on-line resources (*n* = 10). (**b**) The overall workflow of the strategy taken to identify and characterize mantle DEG target genes that regulate shell growth and asymmetry in *M. gigas*. The DEc of the mantle transcriptome of symmetric, slightly asymmetric and asymmetric species was used to identify domains and motifs with the *M. gigas* deduced shell proteins as the reference, and the proteins predicted to be related to shell asymmetry in *M. gigas*. Available *M. gigas* public transcriptome data (normal tissues, larva development stages and shell damage-repair response) was used to predict the probable function of DEnc, and to screen for candidate DEnc related to *M. gigas* shell shape. Mapping *M. gigas* DEc and DEnc in the genome, obtained candidate *cis*-regulatory gene modules, which were analyzed to identify homologue gene modules across five bivalve species. Finally, the function of candidate LncRNA was assessed in gene silencing experiments using siRNA in *M. gigas* with damaged shells and the impact of gene silencing on crystal growth and shell repair rates determined in repaired shells.

### Detection of *cis*-regulatory modules in biomineralization toolbox genes

Mantle protein coding and non-coding DEGs were mapped in the *M. gigas* genome. Ten *M. gigas* chromosomes and two unassembled scaffolds were displayed using the advanced Circos program from TBtools (ver 1.0986) [51] and mapping was done with the chromoMap package (ver 0.3) [52] in R-studio. DEc genes localized 100 kb upstream or downstream of DEnc genes were selected as *cis*-regulation candidates. Detailed mapping of candidate *cis*-regulation modules (DEnc-DEc genes) was performed with the gggenes package (ver 0.4.1) [53] in R-studio. To identify if homologue *cis*-regulation modules were conserved in other bivalves, the *M. gigas* genome was used as a reference and aligned with the *M. yessoensis, P. fucata, M. galloprovincialis, C. virginica* (NCBI Accession: GCA_002022765.4) and *Saccostrea glomerata* (NCBI Accession: GCA_003671525.1) [54] genomes in Diamond (ver 2.0.11, parameters -sensitive; e value <1e^−5^) [55]. Gene synteny and collinearity were identified using MCscan (ver X, parameters s = 5; k = 50; m = 20; e <1e^−5^) [56] and gene synteny plots were visualized in dual synteny plot from TBtools (ver 1.0986) [51]. Orthologues of *M. gigas TIMPDR* and *SMPDR* were identified in *C. virginica* and *S. glomerata* using blastn against the publicly available genome (Supplementary Table 1) and transcriptome (NCBI, Transcriptome Shotgun Assembly, TSA), respectively. The base sequence of the retrieved orthologue regions was aligned using Aliview with MUSCLE (ver 1.24), the percentage of sequence identity was calculated in GeneDoc (ver 2.7) and the non-coding potential confirmed using the defined criteria (indicated above). The candidate gene pairs (DEGnc-DEGc) and their neighbouring gene environments were performed using the gggenes package (ver 0.4.1) [53] in R-studio.

### Shell growth, siRNA experiments and shell damage-repair assays

For the *in vivo* experiments to test the differential growth rates of flat and round valves, juvenile *M. gigas* (*n* = 13/group) were used in a shell damage-repair assay where 3 holes (∼2 mm in diameter) were drilled in the posterior edge of the shell of juvenile *M. gigas*, for details see the SI Appendix. The rational for the assay was based on the predicted differential growth rates that maintain shell asymmetry throughout life in *M. gigas*. Targeted siRNAs were used to ablate specific lncRNA gene expression in juvenile *M. gigas* under a shell damage-repair challenge. The two siRNAs (*TIMPDR* and *SMPDR*) and control siRNA (negative control, NC) provided by GenePharma (Shanghai, China) were diluted in sterile seawater to obtain a working concentration (0.088 μg /μl) and 4.4 μg_(siRNAs)_ /g_(soft body wet weight)_ was injected into the adductor muscle of *M. gigas* in a 50 μl final volume. The concentration chosen was optimized *in vitro* using mantle cell cultures and injections were performed using a micro-syringe (Hamilton Gastight Syringes, Germany) (Supplementary Fig 2). Control (sham, injected with filtered (0.22 μm) sterilized seawater) and siRNA injected *M. gigas* were maintained under experimental conditions and 24 h and 48 h after injections the mantle underneath the damaged flat and round valves of randomly selected animals (*n* = 3) was sampled for molecular expression analysis. The shell damage repair rate of each valve (flat and round, 3 holes/valve, 9 animals, a total of 54 holes /treatment) was determined to assess the effect of gene silencing by measuring the rate of hole repair at day 2. The area of the repaired shell and hole were calculated and a correlation analysis established for growth between the asymmetric valves. The experiments were repeated twice in independent experiments using the same protocol. No animals died during the experiments. To assess if siRNA treatments affected the shell structure the inner surface of the newly grown shells of siRNA treated and control animals were analysed by scanning electron microscopy (SEM), for details see SI Appendix.

**Fig. 2:**
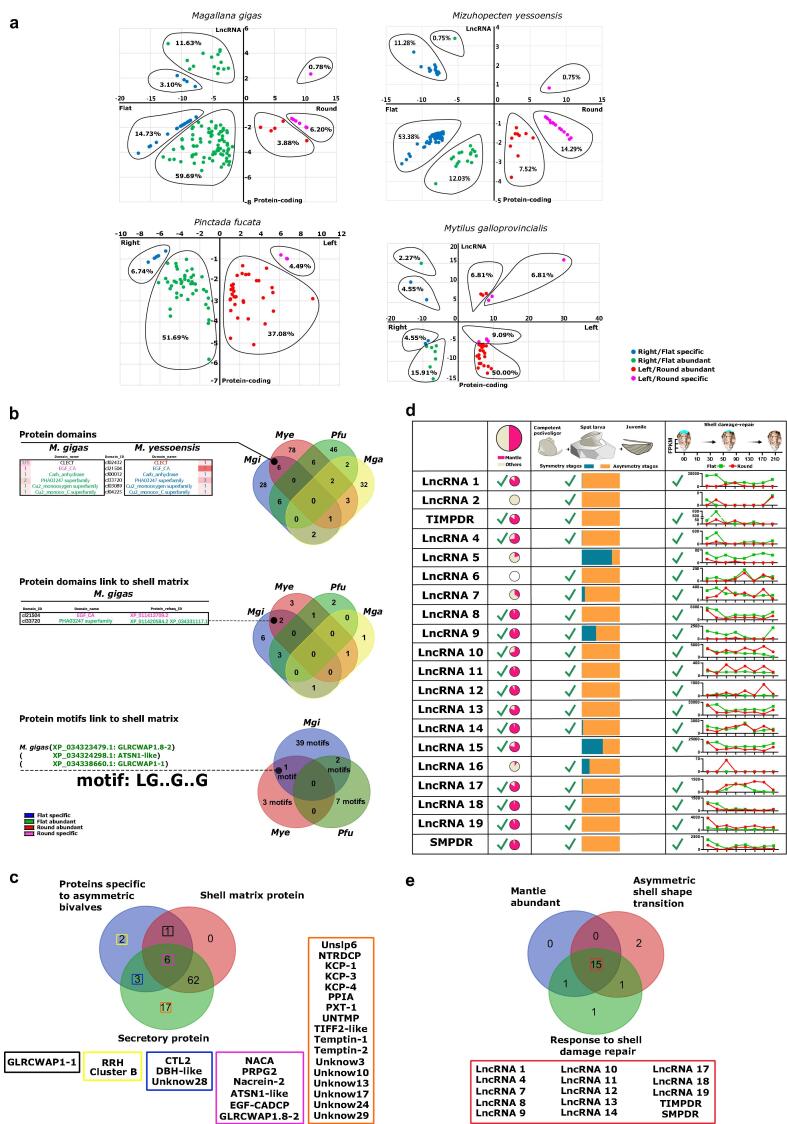
**Potential lncRNA candidates regulating biomineralization toolbox genes in the mantle (selected by their deduced link with asymmetric shell-building).** (**a**). Distribution and expression of DEGs (percentage in relation to all identified lncRNA) in the mantle of two bivalves with asymmetric shells, one bivalve with slightly asymmetric shells, and one bivalve with symmetric shells. The DEGs represented in the graphs for the four species are protein coding and lncRNA. The X-axes represent the significant change (log2 fold change) in DEGs in the mantle of each of the valves (flat or right/ round or left) and the Y-axes represents their abundance and distribution (log2 base mean). Dots are colour coded according to their abundance in the mantle: flat (right valve) is represented in blue (side-specific) and green (abundance) and round (left valve) is in pink (side-specific) and red (abundance). The percentage value indicates the proportion of the mantle DEGs in each species from each valve. (**b**) DEc deduced protein domains and protein motifs common in *M. gigas* and *M. yessoensis* and potentially associated with shell asymmetry. The Venn diagrams shows the mantle specific and overlapping deduced protein domains and SMP-domains and SMP-motifs identified. Domain names and the number of domains identified in deduced proteins are colour coded according to their relative abundance in expressed genes and localisation in the mantle of the round or flat valves. The protein motif LGXXGXXG (where X represent any amino acid residue) was found in both *M. gigas* and *M. yessoensis*. In *M. gigas* three DEc genes with an LGXXGXXG motif were found and were most abundant and enriched in the mantle transcriptome of the flat valve. (**c**) The Venn diagram shows the number and overlap of the DEc genes identified using the following criteria: 1) secretory proteins (green), 2) putative SMPs (red), 3) proteins with domains specific to the asymmetric bivalve mantle (blue). (**d**) Expression and distribution profile of the selected DE lncRNAs (lncRNAs 1 to 20) in the *M. gigas* mantle transcriptome of the flat and round valve. The pie chart represents the relative abundance in the mantle transcriptome in relation to other tissues. When transcript abundance was > 70 % in the mantle they were considered as strong candidates for biomineralization “√”. The rectangular chart represents the relative abundance in the asymmetric stages in relation to other symmetric stages. When transcript abundance was > 70 % in the asymmetric stages they were considered as strong candidates for asymmetric shell biomineralization “√”. Expression during shell damage-repair experiments (duration 21 days) in adult *M. gigas*. Transcript expression data for the mantle of the flat and round valves are represented by green and red lines, respectively. Transcripts marked “√” were abundant and responded to shell damage-repair and were selected for further analysis. (**e**) The Venn diagram shows the number and grouping of lncRNAs by 1) regulated response to shell damage-repair (green), 2) regulation in larval stages associated with shell shape transition (red), 3) expression abundance in the mantle (blue).

### Statistical analysis

For the statistical approaches used for omics-data see the relevant methods section. For other experimental treatments significant differences (*p* < 0.05) between groups were calculated with a two-tailed Student’s *t*-test using GraphPad Prism ver 8.0.2. Data are presented as the mean ± SEM.

## Results

### Differentially expressed genes (DEGs) are more abundant in bivalve flat valve mantle transcriptomes

Mantle transcriptomes from *M. gigas* (asymmetric shell, this study) and *M. galloprovincialis* (symmetric shell, this study) and from two other bivalve species *M. yessoensis* (asymmetric shell), *P. fucata* (slightly asymmetric shell), where mantle transcriptomes of both valves and an annotated genome exist, were used to identify differentially expressed genes (DEGs) in the mantle (Fig. 1**,** Supplementary Table 1, 2). The correlation between intraspecies samples for each of the transcriptomes analysed was more than 0.8, indicating that the transcriptomes were of similar high-quality (Supplementary Fig 3). In *M. gigas*, a total of 129 DEGs were identified (∼0.4 % of the predicted genes in the species genome), of which 115 were unique to, or more abundant in, the mantle of the flat valve. In *M. yessoensis*, 133 DEGs were found of which 103 were unique to, or more abundant in, the mantle of the flat valve. In the mantle of the slightly asymmetric shell of *P. fucata*, 89 DEGs were identified, of which 37 DEGs were more abundant in the left mantle and 52 in the right mantle. But in the mantle of the symmetric shell of *M. galloprovincialis,* 44 DEGs were identified, of which 32 DEGs were more abundant in the left mantle and 12 in the right mantle (Supplementary Fig 3**,** Supplementary Table 3). In *M. gigas*, *M. yessoensis* and *M. galloprovincialis*, DEG protein coding and lncRNA genes were identified (Fig. 2**a**).

**Fig. 3:**
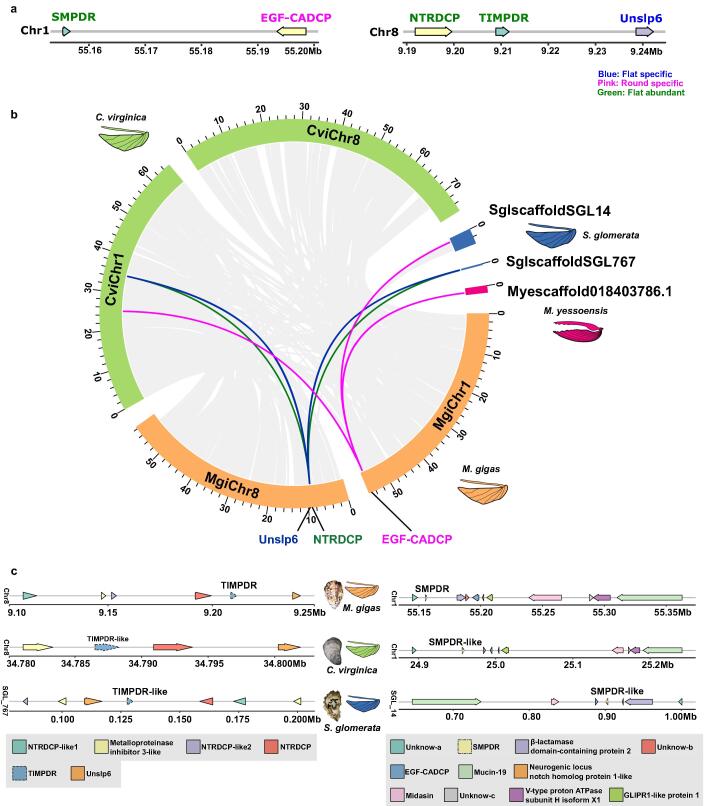
**Mantle lncRNAs and candidate *cis*-regulation modules in bivalve/Ostreidae family.** (**a**) Two candidate DEG *cis*-regulatory modules were selected in *M. gigas*. Arrows indicate gene orientation and gene position is provided (Megabases, Mb). Protein coding gene symbols and lncRNAs are indicated and are coloured according to distribution and abundance in the *M. gigas* mantle. The candidate *cis*-regulatory module of *TIMPDR* included uncharacterized shell protein 6-like (*Unslp6*) and NTR domain-containing protein-like (*NTRDCP*), which encoded TIMP domain mantle proteins, and are likely specific to *M. gigas*. The candidate *SMPDR cis*-regulatory module contained the gene *EGF-CADCP* (EGF-CA domain containing protein), that codes for an SMP and was common in the mantle transcriptome of the asymmetric bivalves studied. (**b**) Collinearity between *M. gigas* and homologue genome regions in other bivalves. Lines (grey) represent gene blocks with a minimum of five orthologue genes and lines (blue, green and pink) indicate collinear gene blocks that contain the candidate protein coding genes. Candidate protein coding genes are mapped to *M. gigas* chromosome 1 (*EGF-CADCP*) and chromosome 8 (*NTRDCP* and *Unslp6*) and homologous collinear genome regions were found in *C. virginica* (chr 1 and 8) and in *S. glomerata* SGL_14 and SGL_767, respectively. No homologue regions containing the target coding genes were found in the genomes of *P. fucata* and *M. galloprovincialis* (slightly asymmetrical/symmetrical bivalves, Supplementary Fig 19) or the scallop *M. yessoensis* (asymmetrical bivalve) but a gene with a similar protein domain to *EGF-CADCP* (EGF_CA domain) was identified in the scallop genome, scaffold NW_018403786.1. (**c**) The *M. gigas* genes were taken as a reference to identify homologue genome regions in other members of the Ostreidae family with an asymmetric shell. Detailed characterization of the collinear gene blocks (>five homologue coding genes) containing the *M. gigas* candidate DEc genes and the lncRNAs for the homologue genome regions in the Ostreidae family members analysed, *C. virginica* and *S. glomerata* (see Supplementary Table 5). Lines represent the genome regions and arrows represent genes and arrow heads indicate gene direction according to the genome annotation. Arrow length is related to gene length. Gene names and their relative position in the genome (Mb) are indicated. Orthologue genes are indicated in the same colour and the gene symbol is given. The lncRNA genes analysed are represented by dashed arrows.

### Asymmetric expression of biomineralization toolbox genes in the mantle

DEGs encoding secreted proteins in the mantle associated with the flat or round valves corresponded to 80.73 % in *M. gigas*, 15.52 % in *M. yessoensis*, 45.45 % in the slightly asymmetric *P. fucata* and 37.08 % in the symmetric *M. galloprovincialis* (Supplementary Fig 4**,** Supplementary Fig 5). In *M. gigas*, DE genes encoding proteins with a whey acidic protein (WAP, cl00156) domain were most abundant and were highly represented in the mantle transcriptome of the flat valve and in *M. yessoensis*, genes encoding proteins with a Calcium-binding Epidermal Growth Factor-like domain (EGF_CA, cl21504) were most abundant and were highly represented in the mantle transcriptome of the flat valve (Supplementary Fig 6). The WAP domain in proteins has been proposed to inhibit crystal growth in the fast-growing c-axis and form platy geometrical aragonite crystals [57]. In *P. fucata*, genes encoding proteins of Solute carrier families 5 and 6-like; solute binding domain (SLC5-6-like_sbd, cl0045) were abundant in the mantle transcriptome of the left valve. SLC5-6-like_sbd domain functions as an ion transporter and is a member of the biomineralization toolbox genes. In *M. galloprovincialis,* proteins with a Low Density Lipoprotein Receptor Class A domain (LDLa, cl00104), that are proposed to function as cell-surface receptors, were most abundant and were highly represented in the mantle transcriptome of the left valve. Comparison of the encoded proteins for the DEGs in the four species analysed indicated that six protein domains were restricted to the DEGs of *M. gigas* and *M. yessoensis* [C-type lectin domain (CLECT), EGF_CA, Carbonic anhydrase (Carb_anhydrase), PHA03247 superfamily, Copper type II ascorbate-dependent monooxygenases (Cu2_monooxygen) superfamily, Copper type II ascorbate-dependent monooxygenase C-terminal domain (Cu2_monoox_C) superfamily], and a bias in their expression between the mantle transcriptomes of the two valves was a characteristic of completely asymmetric valves as they were absent from *P. fucata* and *M. galloprovincialis* DEGs (Fig. 2**b**).

**Fig. 4:**
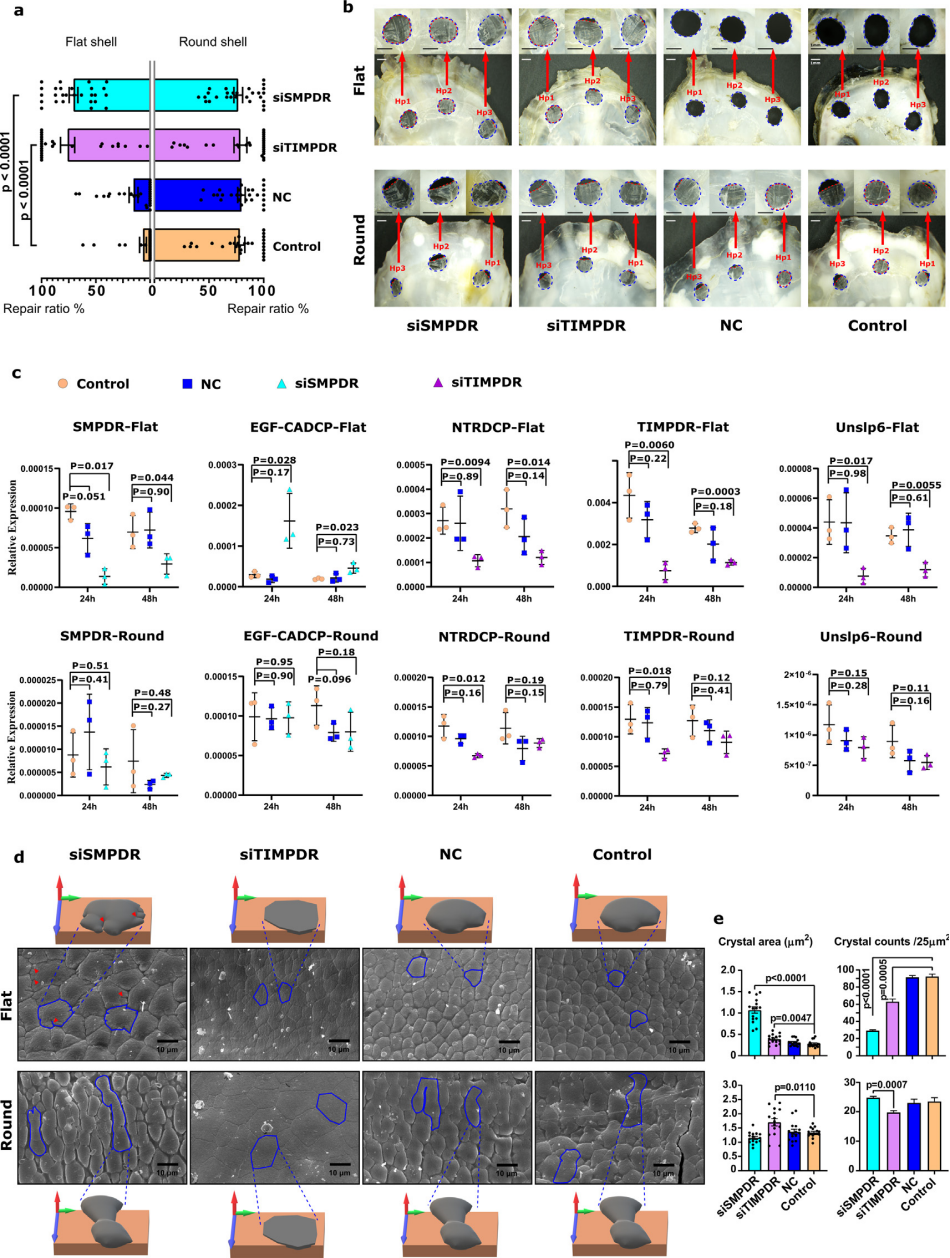
**Effect of lncRNAs on mantle gene expression, shell damage-repair and shell structure of both valves in juvenile *M. gigas*.** (**a**) Shell repair ratio in the flat and round valves 2 days after drilling: Control (seawater, no siRNA chain), negative siRNA chain (NC) and the two treatments groups (siRNA-*TIMPDR* and siRNA-*SMPDR*). Data is presented as the median and quartile ranges from 9 juvenile *M. gigas* for each treatment group (Control, NC, siRNA) where each valve (flat and round) was drilled with 3 holes (6 holes per animal, 54 holes in total/treatment). (**b**) Bright field digital photographs showing the recovery of the newly produced shell 2 days after drilling in juvenile *M. gigas* flat and round valves. A representative image from one individual/ experimental group is shown and was captured using a stereoscope (Motic, SMZ-171, China) equipped with a digital camera (Visicam 6 Plus, VWR, Portugal). Detailed magnification of the three-hole positions (Hp1, Hp2 and Hp3) is shown. The red-dashed line indicates the limit of the newly grown shell in each hole. The scale bar corresponds to 1 mm. (**c**) Effect of shell damage on mantle gene expression. Data was obtained by quantitative expression (q-RT-PCR) and is shown as mean ± SEM, (*n* = 3 pooled samples: 3 individuals/pool). Significant differences in gene expression were calculated by comparison to the damaged group and control group using a Student *t*-test. The p values for the pairwise comparisons are shown. (**d**) Scanning electron microscope (SEM) images of the prismatic calcium carbonate crystals of the inner side of the recovered flat and round valves. Representative prismatic crystals are delineated with a blue line to highlight their structure and an illustrative 3D model of their structure was designed using Paint 3D (Microsoft, USA). The red arrowheads indicate slits at the periphery of calcium carbonate crystals in the flat valve of the siRNA-*SMPDR* group. (**e**) Prismatic crystal area of shell-repair in the flat and round valves was calculated from: i) the areas of randomly selected crystals (*n* = 15) or ii) the number of crystals present in a randomly selected 25 mm^2^ area (*n* = 4).

**Fig. 5:**
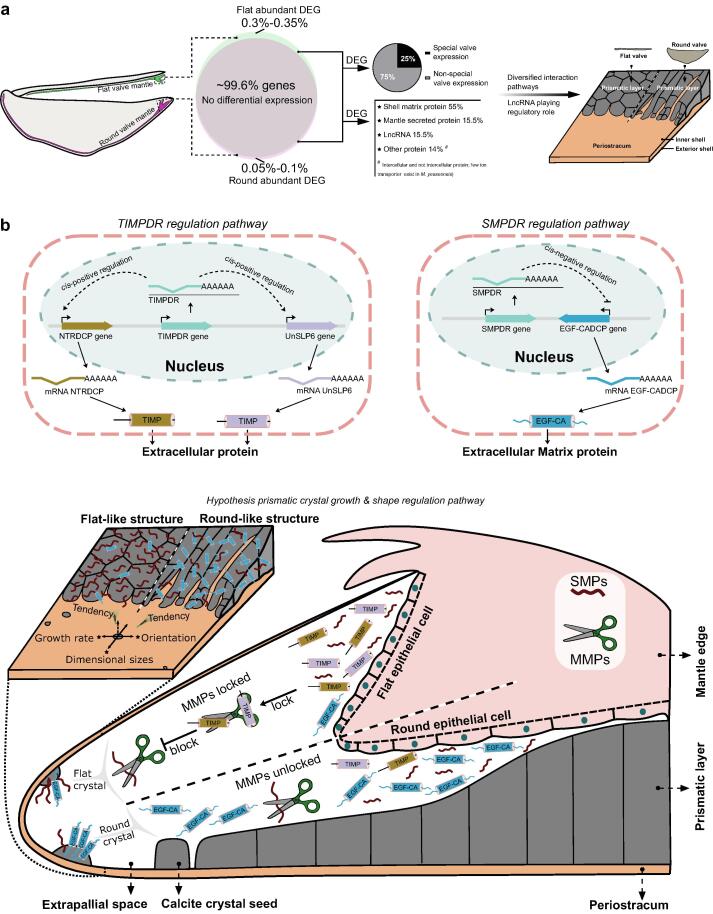
**Putative regulatory model explaining the production of asymmetric shells in oyster.** (**a**) The shell shape is regulated by a very small number of DEGs in the mantle margin, the majority of which are biomineralization toolbox genes, and include lncRNA, and genes coding for secreted and intracellular proteins. Most DEGs are specific to the flat valve and twenty-five percent have valve-specific expression. (**b**) A putative model to explain how lncRNAs *TIMPDR* and *SMPDR* affect the expression of neighbouring protein coding genes. *TIMPDR* positive *cis*-regulation of the secreted non-matrix proteins *Unslp6* and *NTRDCP*, which contain a TIMP domain, explains their abundant expression in the mantle of the flat valve. *SMPDR* negative *cis*-regulation of the *EGF-CADCP* matrix protein (EGF-CA domain containing), which is abundantly expressed in the mantle of the round valve. It is hypothesized based on the siRNA experiments and SEM results for the prismatic calcium carbonate that i) expression of the SMP EGF-CA domain promotes the growth and area (shape) of the prismatic crystals in the round valve and b) expression of the TIMP domain protects SMPs from matrix metalloproteinases (MMP) and stimulates prismatic shell growth in the flat valve. The resulting differences in growth rate and shape of prismatic crystals causes shell asymmetry in oysters.

To identify additional SMPs, *M. gigas* mantle transcriptomes were compared to their shell proteome (Supplementary Fig 7**a**). A total of 12 SMP-domains [WAP, Tyrosinase, GH20_hexosaminidase superfamily, Glycoside hydrolase family 20 (GH20_hydro_20b) superfamily, putative carbohydrate-binding domain (CHB_HEX) superfamily, EF-hand calcium binding motif (EFh) superfamily, von Willebrand factor type C (VWC), EGF_CA, Lytic polysaccharide mono-oxygenase cellulose-degrading (LPMO_10), PHA03247 superfamily, PRK10263 superfamily, Topoisomerase II-associated protein PAT1 (PAT1) superfamily] were found in deduced proteins of the mantle transcriptome. Most of the proteins identified by proteomics were abundant or specific to the mantle transcriptome of the flat valve except the EGF_CA domain which was specific to the mantle transcriptome of the round valve (Supplementary Fig 7**b**). Only two SMP-domains (EGF_CA, PHA03247) were encoded by genes identified in the transcriptomes of *M. gigas* and *M. yessoensis* (Fig. 2**b**). *In silico* analysis of gene transcripts also identified a novel SMP-motif “LGXXGXXG” (X represents any amino acid residue), present in asymmetric bivalves and abundantly expressed in the mantle of the flat valve (Supplementary Fig 8-9). Five groups of DEc genes with potential candidate functions in shell shape [GLRCWAP1-1 (group1); RRH and Cluster B (group2); CTL2, DBH-like and Unknow 28 (group3); NACA, PRPG2, Nacrein-2, ATSN1-like, EGF-CADCP and GLRCWAP1.8–2 (group4); Unslp6, NTRDCP, KCP-1/3/4, PPIA, PXT-1, UNTMP, TIFF2-like, Temptin-1/2 and Unknow 3/10/13/17/24/29 (group5)] were identified that complied with the criteria “secretory protein”, “putative SMP”, and “proteins with domains specific to the asymmetric bivalve mantle” (Fig. 2**c**). Overall, 66.06 % of *M. gigas* DE coding genes (DEc) were potential SMPs, of which 62.39 % were abundant or specific to the mantle of the flat valve (Supplementary Fig 10**a**) but only one DEc gene in *M. galloprovincialis* encoded an SMP. Genome mapping revealed that DE transcripts in *M. gigas* were widely distributed across the 10 chromosomes but were relatively more abundant on chromosome 2 and 3 (Supplementary Fig 10**b**).

### Asymmetric expression of long non-coding RNA genes in the mantle

LncRNAs accounted for 15.50 % of DEGs in *M. gigas* and 12.78 % in *M. yessoensis* and were most abundant in the mantle of the flat valve (Fig. 2**a**). LncRNAs accounted for 19.44 % of DEGs in *M. galloprovincialis* and were more abundant in the mantle of the left valve. LncRNAs have been neither predicted nor documented in the latest release of the genome assembly of *P. fucata* [48] and so were not analysed in this study (Fig. 2**a**). Twenty-one lncRNAs were predicted in the genome of *M. gigas* and 20 were confirmed as authentic (lncRNA 1–20, Supplementary Table 4) and 19 were considered abundant or specific to the *M. gigas* flat valve mantle transcriptome. To select candidate lncRNA for functional studies publicly available mantle transcriptomes (Supplementary Table 1**,** Supplementary Fig 11**a**), including transcriptomes of larval stages associated with shell/shape establishment (Supplementary Fig 11**b**), and transcriptomes of shell damage-repair (Fig. 2**d**) were analysed and fifteen potential candidate lncRNAs associated with completely asymmetric shell-building were identified [*lncRNA 1*, *lncRNA 4*, *lncRNA 7*–*14*, *lncRNA 17*–*19*, *TIMPDR* and *SMPDR*] (Fig. 2**e**).

### Long non-coding RNAs are regulators of biomineralization toolbox genes

Six LncRNAs were assigned to potential *cis*-regulatory modules (Supplementary Fig 12**a**). Based on transcriptome evaluation, four candidate lncRNAs [*lncRNA 1*, *lncRNA 17*, TIMP (tissue inhibitor of metalloproteinases) domain regulator (*TIMPDR)*, SMP domain regulator (*SMPDR*)] were selected as likely regulators of DEc genes in the mantle (Supplementary Fig 12**b**). *LncRNA 1* and *lncRNA17* were excluded from further analysis because of high sequence similarity with the Acanthoscurrin-1-like (*ATSN1-like*) gene and extensive highly repetitive regions in the downstream regulatory protein, respectively, which precluded specific primer design.

The candidate *cis*-regulatory module of *TIMPDR* included uncharacterized shell protein 6-like (*Unslp6*) and NTR domain-containing protein-like (*NTRDCP*), which encoded TIMP domain mantle proteins. The candidate *SMPDR cis*-regulatory module contained the gene *EGF-CADCP* (EGF-CA domain containing protein), that codes for an SMP (Fig. 3**a,** Supplementary Fig 13). Expression analysis of *cis*-regulation module genes was evaluated using *in vitro* cultures of mantle tissue (Supplementary Fig 14).

Collinear *cis*-regulatory regions were conserved in the evolutionary proximate asymmetric shell species *C. virginica* and *S. glomerata* (Fig. 3**b–c,** Supplementary Fig 15). No corresponding collinear regulatory modules were identified in the genomes of the asymmetric shelled *M. yessoensis* or slightly asymmetric shelled *P. fucata* or in the symmetric shelled *M. galloprovincialis*.

### Non-coding RNAs regulate shell growth and crystal structure in oyster

To test the potential role of candidate lncRNAs on shell biomineralization, shell damage-repair assays in juvenile *M. gigas* were used. Shell damage-repair assays revealed that in juvenile *M. gigas* repair of damage to the round valve was faster than repair in the flat valve. Two days after shell perforation, 80.05 ± 20.82 % of the 3 holes in each shell had been covered in the round valve compared to 12.84 ± 20.19 % in the flat valve (*n* = 13, *p* < 0.00001, *Z* = 14.48 (*z*-test), Supplementary Fig 16). To test the role of lncRNA in shell biomineralization, small interfering RNAs (siRNA) were applied in *M. gigas* damage-repair trials. The oysters of the control (no lncRNA, only seawater) and NC (negative control, lncRNA control chain) experimental groups had a similar shell repair rate (mean value in round and flat valves were 79.71 % and 15.26 %, respectively) but the shell repair rate was altered in the siRNA-lncRNA treated groups. The damage-repair ratio in the round and flat valves of the group treated with siRNA-*TIMPDR* was similar (78.77 ± 25.48 % and 76.09 ± 28.82 %, respectively), and represented a significant increase in biomineralization of the flat valve compared to the normal flat valve repair rate in the control group (*p* < 0.001). Similarly, in siRNA-*SMPDR* treatments, flat valve repair rates (70.65 ± 20.87 %) were significantly greater than in the negative control lncRNA (*p* < 0.001) (Fig. 4**a, b**) groups. In the flat valve, siRNA-*TIMPDR* also caused a significant reduction (*p* < 0.05) in the expression of the target genes, *NTRDCP* and *Unslp6*, whereas ablation of *SMPDR* significantly up-regulated *EGF-CADCP* gene expression (*p* < 0.05) (Fig. 4**c**). The results confirmed that the candidate lncRNAs affected shell repair of the flat valve by modifying expression of mantle and SMP genes.

The newly grown shell of the flat and round valve of the siRNA-*TIMPDR* oysters had a similar prismatic layer organization, which differed from the control groups (control and NC) (Fig. 4**d**). In both valves, the boundaries between the crystals were narrower and the calcium carbonate crystals were flattened, had lost their typical convex shape and were larger than the normal crystals of the control groups (Fig. 4**d–e，**Supplementary Fig 17). The newly grown round inner shell valve of siRNA-*SMPDR* oysters, also modified shell structure, causing the prismatic crystals in the round valve to become larger without changing their shape compared to the control. In the flat shell the prismatic crystals were irregular with fissures and a horizontal orientation (Fig. 4**d–e**).

## Discussion

Shell biomineralization is tightly regulated by the mantle and haemocytes, and although some biomineralization toolbox genes are conserved across bivalves, its utilization for shell building is divergent across species and lineage-specific mantle secretomes are considered to underlie bivalve shell diversity and shape [25], [58]. Modern oysters and scallops, possess completely asymmetric shell valves and an asymmetric shell shape is acquired early in bivalve larval development when differential gene regulation begins [59], [60]. Our *meta*-analysis of bivalve transcriptomes and proteomes suggests that asymmetric shell development is related to a small proportion of differentially expressed SMP genes that are most actively expressed by the mantle producing the flat valve. The divergent gene expression between the mantle of the flat and round valves is orchestrated by a set of conserved non-coding RNAs that modify shell growth and the spatial organization of the crystals (Fig. 5**a**).

Despite the existence of relatively limited experimental evidence linking lncRNA to biological function in bivalves, previous transcriptome/genome screening identified them in the mantle, and they have been implicated in the regulation of shell pigmentation, larval development, the immune system, and shell formation [35], [36], [61], [62], [63], [64]. In bivalves, RNAi knockdown experiments indicated that lncRNAs regulate immune gene (IL-17) expression [37] stimulated by poly I:C [39]. In *P. fucata,* expression of lncMPEG1 was stimulated by shell damage, alien invasion and temperature and hypoxia stresses. Furthermore, decreased lncMPEG1 expression is associated with irregular crystal growth on the inner surface of the prismatic layer and nacre in *P. fucata* shells [65]. In addition, RNAi knockdown experiments in *P. fucata* demonstrated that lncMSEN2 is related with the immune response and regulation of nacreous layer formation [40]. None of the previous studies about lncRNA in bivalves considered their role in the regulation of shell growth or shape. In the present study we provide evidence that lncRNAs most likely regulate shell growth and shape in adults. Furthermore, we used publicly available transcriptomes from *M. gigas* early larval development stages and during the transition from a larva with symmetrical valves (free-swimming competitive pediveliger larvae) to a larva with asymmetrical valves (sessile spat) after metamorphosis to identify candidate genes involved in emergence and establishment of the shell. In bivalves, the shell emerges during the first stages of development and in *M. gigas* the shell gland starts to secret the shell 24 h post-fertilization (D-shape larvae) [66], [67]. After the shell is formed, the larvae develop into pediveliger larvae the last stage before settlement on a hard substrate and metamorphosis into a sessile juvenile spat with an asymmetric shell [68], [69]. During larval stage transitions, the morphology, biochemistry, and composition of the calcifying tissue of oyster larvae changes and most studies of this ontogenetic stage have identified genes including lncRNA that change in expression and may be related to shell development [36], [50], [63], [70]. In the present study by conducting *meta*-analysis of data from larvae developing a shell and the mantle from adults, novel biomineralization candidate genes and regulatory lncRNAs that regulate shell growth were identified. Although future studies are required to confirm binding partners and their common spatial–temporal expression and experimental confirmation that they orchestrate shell development and growth in larvae. The involvement of lncRNAs in insect metamorphosis has been described and our results provide further support for the involvement of lncRNA during invertebrate development and emphasise their likely importance in Mollusca [71], [72].

The differential distribution of lncRNAs between the mantle of flat and round valves, their proximity to SMP loci and the conservation of the *cis*-regulatory modules in other asymmetric oyster species indicates this regulatory mechanism probably emerged in the common ancestor of this taxonomic group. Using *M. gigas* as the model, we found that the number of DEGs between the mantle of the flat and round valves are relatively small, and differential gene regulation is likely to explain divergent rates of valve growth. To test if lncRNA modulated DEG expression and modified shell growth, small interfering RNAs (siRNA) were applied in *M. gigas* shell damage-repair trials. The results obtained confirmed that the candidate lncRNA (*TIMPDR* and *SMPDR*) affected shell repair through modified expression of mantle (*NTRDCP* and *Unslp6*) and SMP (*EGF-CADCP*) genes.

The diversity of calcified shell types, despite the conserved “building blocks”, in extant bivalves is due to the deposition of crystalline and amorphous calcite crystals in an organic framework [73], [74] and the shell of asymmetric bivalves usually contain different microstructures [75]. In *M. gigas* the flat and round valves have a different crystal structure [23], [76]. The prismatic crystals of the flat shell have a near-geometric crystal structure with regular boundaries and no cracks resembling the prismatic structure of symmetric shells [77]. In contrast the crystals of round shells have a ridge-and-furrow structure, which is associated with their rapid growth for substrate adherence [23]. The explanation for the divergent shell growth and modified shell architecture in the asymmetric shell compared to the symmetric shell of the oysters (Pteriomorphia) may be explained by the unique bias in the expression of some biomineralization toolbox genes in the mantle of the asymmetric shells and the evolution of shared colinear *cis*-regulatory modules. When this is considered in the context of the vast diversity of bivalve species, and their complex phylogenetic relationships the consensus view is that the modern bivalves typically display bilateral symmetry both in shell and anatomy and only a few bivalves such as oysters (Ostreidae), some clams (Anomiidae and Chamidae) and scallops (Pectinida) acquired valve asymmetry during evolution [6], [75], [78], [79], [80]. We propose based on the results of our study that the symmetry and round shells in bivalves is the default condition and that the flat valve of the asymmetric oysters is a more recent evolutionary innovation.

The new growth of damaged shells of both the flat and round valves in siRNA-*TIMPDR* treated *M. gigas* had a similar prismatic layer organization. To understand why modified expression of the non-SMPs, *Unslp6* and *NTRDCP*, affected the shell we analysed the characteristics of the deduced proteins. Both contained a TIMP domain and belonged to the NTR-like superfamily that has inhibitory matrix metalloproteinase activity in vertebrates [81] and in *P. fucata* [82] (Supplementary Fig 18**,** Supplementary Table 6). In *P. fucata* metalloproteinases degrade extracellular matrices to produce the fine organic fibres that regulate the orientation of fibrous aragonite crystals [83]. siRNA-*SMPDR* also modified shell structure, causing the prismatic crystals in the round valve to become larger, although their shape was not changed and was similar to the control. The EGF domain containing protein in Pinctada shell was reported to only exist in the prismatic layers [84], which implies that this domain may have an important role in specific crystal nucleation events and arrangements [9], [85]. In the flat valve siRNA-*SMPDR* caused the prismatic crystals to be irregular with fissures and a horizontal orientation. The negative regulation of *EGF-CADCP* by *SMPDR* also had a positive effect on rapid planar growth of prismatic calcium carbonate crystals.

In summary, in our study we identify a conserved suite of enriched protein domains and regulatory factors of bivalve shell growth and propose a model to explain the origin of asymmetric valves in oysters and possibly other asymmetric bivalves. We propose that modified expression of SMP and non-SMP proteins regulates growth of prismatic calcium carbonate crystals and thus the shell (Fig. 5**b,** Supplementary Table 7). Our results indicate that low expression of *SMPDR* in the mantle of the round valve upregulates the expression of EGF-CADCP (SMP), we hypothesise that this affects the deposition of secreted protein in the prismatic layer and promotes shell growth and the formation of crystals of calcium carbonate with an irregular spatial orientation. In contrast, high expression of *TIMPDR* in the mantle of the flat valve upregulates NTRDCP and Unslp6 (non-SMPs) expression, which we hypothesise increases secretion of proteins with a TIMP domain into the extrapallial fluid. High concentrations of TIMP domain proteins are likely to inhibit matrix metalloproteinase activity in the extrapallial fluid and decrease degradation of SMPs to promote slower shell growth and a regular spatial orientation of the prismatic crystals.

In conclusion, we have demonstrated that valve asymmetry in oyster originates from the differential expression of some genes in the mantle of flat and round valves as a result or regulation by lncRNAs. The *cis*-regulatory modules identified explain the asymmetric expression in the mantle of some biomineralization-related SMP and secreted non-SMP genes that modulate prismatic crystal growth, by affecting the planar growth rate and spatial orientation of crystals in shells. The present study in oyster substantiates the hypothesis that shells are shaped by conserved upstream *cis*-regulatory factors and a highly evolved and diverse downstream regulatory network in the bivalve mantle [25], [29].

**Compliance with Ethics Requirements**

All Institutional and National Guidelines for the care and use of animals (fisheries) were followed.

## Declaration of Competing Interest

The authors declare that they have no known competing financial interests or personal relationships that could have appeared to influence the work reported in this paper.
