# Supplementary material for: Core genes of biomineralization and cis-regulatory long non-coding RNA regulate shell growth in bivalves
Source: J Adv Res. 2023 Nov 22;64:117–29. doi: 10.1016/j.jare.2023.11.024 (PMC11464482; doi:10.1016/j.jare.2023.11.024)
Supplement: Supplementary data 1 [file mmc1.docx]

**SUPPORTING INFORMATION (SI)**

**Core genes of biomineralization and cis-regulatory long non-coding RNA regulate shell growth in bivalves**

Maoxiao Peng^1^, João C.R. Cardoso^1*^, Gareth Pearson^2^, Adelino VM Canário^1,3,4^ and Deborah M. Power*^1,3,4^

^1^ Comparative Endocrinology and Integrative Biology, Centre of Marine Sciences, Universidade do Algarve, Campus de Gambelas, 8005-139 Faro, Portugal

^2^ Biogeographical Ecology and Evolution, Centre of Marine Sciences, Universidade do Algarve, Campus de Gambelas, 8005-139 Faro, Portugal

^3^ International Research Center for Marine Biosciences, Ministry of Science and Technology, Shanghai Ocean University, Shanghai, China

^4^ Key Laboratory of Exploration and Utilization of Aquatic Genetic Resources, Ministry of Education, Shanghai Ocean University, Shanghai, China

**MATERIALS & METHODS**

**Isolation of cell nuclei and cytosol from the mantle**

To determine the subcellular localization of *TIMPDR* and *SMPDR*, in the mantle of *M. gigas*, the nuclear/cytoplasmic RNA fractions were isolated as described in Sui et al. 2020 (1) with minor modifications. The mantle collected from the flat or round valves (≈ 50mg, n = 3) in adult *M. gigas* was washed twice in PBS (137 mM NaCl, 2.7 mM KCl, 8 mM Na_2_HPO_4_, and 2 mM KH_2_PO_4_; pH 7.4) and homogenized with a hand-held pestle and mortar in 1 ml of buffer A (10 mM HEPES (pH 7.9), 1.5 mM MgCl_2_, 10 mM KCl, 1 mM EDTA, 1 mM DTT, 0.05% NP-40, and RNase inhibitor, Thermo, EO0381) for 1 min. Homogenates were left on ice for 15 min, and the nuclei separated from the cytosolic fraction by centrifugation at 600 x g at 4 °C for 10 min. Nuclei were washed in buffer A, and total RNA (tRNA) was extracted as described below. The supernatant from the isolation of nuclei was centrifuged for 15 min at 20,000 x g, 4°C to remove cellular debris before extraction of tRNA.

**Total RNA extractions**

Total RNA (tRNA) of *M. gigas* and *M. galloprovincialis* was extracted using an E.Z.N.A Total RNA Kit I (Omega, USA) following the manufacturer’s protocol, and genomic DNA contamination was removed using Precision DNase (Primer design, UK). The amount and quality of tRNA extracted was assessed by absorbance (NanoDrop, Thermo Scientific, USA) and by electrophoresis on 1% TAE agarose gels. For the transcriptome libraries the tRNA from the mantle of six individuals were extracted and the concentration measured using a Qubit RNA Assay Kit in a Qubit 2.0 Fluorometer (Life Technologies, CA, USA). RNA integrity was assessed using an RNA Nano 6000 Assay Kit for a Bioanalyzer 2100 system (Agilent Technologies, CA, USA) and samples that passed a preestablished threshold for the RNA integrity number (RIN > 8.0) were used for RNA library preparation (n = 3 pools, two individuals/pool).

**Small scale gene expression analysis**

***cDNA synthesis for quantitative-RT-PCR***

All cDNAs were synthesized from DNase treated tRNA (500 ng) denatured at 65ºC for 5 min and quenched for 5 min on ice. Reactions were carried out in a 20 μl final volume with 10 ng of pd(N)6 random hexamers (Jena Bioscience, Germany), 2 mM dNTPs (ThermoScientific, USA), 100 U of RevertAid Reverse Transcriptase and 8 U Ribolock RNAse inhibitor (ThermoScientific) and the reaction conditions were: 10 min, 20 ºC; 60 min, 42 ºC; 70 ºC, 5 min. For the confirmation of mantle transcriptome gene expression, mantle cDNA from flat and round valves of *M. gigas* was prepared from 5 individuals. For other expression studies (SMP gene quantification, lncRNA abundance, knock-down studies) each cDNA was prepared from a pool of tRNA with equal contributions from two individuals and three samples were prepared from six individuals (n = 3 pools, two individuals/pool).

***Quantitative-RT-PCR (qRT-PCR)***

Q-RT-PCR reactions were performed using SsoFast EvaGreen Supermix (Bio-Rad, Portugal) for a 10 µl final reaction volume containing 200 nM of candidate gene specific primer pairs (**Supplementary Table 8**) and 2 µl of cDNA template (diluted 1:2). Duplicate reactions were performed (and < 5% variation accepted between replicates) and all reactions were run on a CFX Connect Real-Time PCR Detection System for 96-well microplates (Bio-Rad). Melting curves were performed to detect non-specific products and primer dimers. Reverse transcriptase (RT-) and PCR control (no template) reactions were included in all qRT-PCR assays to confirm the absence from samples of genomic DNA or contamination, respectively. Reaction efficiencies and R^2^ (coefficient of determination) were established for each primer pair. The *M. gigas* elongation factor 1-alpha (EF1α) and the ribosomal protein L7 (RL7) were used as reference genes for normalization (cDNA diluted 1:100 and 1:10, respectively). The expression of EF1α and RL7 between the samples were relatively constant and target gene expression was normalized using the geometric mean of the expression level of the two reference genes.

**Transcriptome quality control, assembly and calculation of differentially expressed genes**

*M. gigas* and *M. galloprovincialis* sequencing libraries were prepared with an Illumina TruSeq stranded mRNA-seq library Prep kit (RNA input 2 μg, insert size of 300–400 bp) and sequencing performed on Illumina Hi-Seq 2000 (for *M. gigas*) and Illumina NovaSeq 6000 (for *M. galloprovincialis*) and 150 base paired-end reads were generated. Transcriptomes of the *M. gigas* and *M. galloprovincialis* mantle from the round/left and flat/right valves were analyzed in Galaxy (https://usegalaxy.eu/) and annotated in R-studio. The quality of the transcriptome was assessed using FastQC (Version 0.72) with the default parameters (2) and Trimmomatic (Version 0.36.5) was used to trim low quality reads (3). Clean reads of *M. gigas* and *M. galloprovincialis* were mapped to the *M. gigas* annotated reference genome (NCBI Accession: GCA_902806645.1) and *M. galloprovincialis* annotated reference genome (NCBI Accession: GCA_900618805.1), respectively, using HISAT2 (Version 2.1.0, default settings) (4) and transcript counts generated with StringTie (Version 1.3.6, default settings) (5). Correlations between the output obtained for biological replicates was performed with the corrplot tool in R-studio (version 0.90, default settings) (6). Gene expression (FPKM) was calculated with a spearman correlation coefficient. Identification of DEGs was performed in R-studio using DESeq2 package (7) with an FDR <0.05, log2FC >1 or log2FC<-1, respectively. Statistical validation was performed using Volcano plots constructed in the ggplot2 package (8) using the same threshold as in DESeq2 and heatmaps were constructed using the pheatmap package (9). The Sequence Read Archive (SRA) accession number of the raw data from *M. gigas* and *M. galloprovincialis* is PRJNA882340. qRT-PCR for 10 genes from *M. gigas* was used to verify the accuracy of the *M. gigas* transcriptome data. The differential expression determined by qRT-PCR (2 DEnc and 8 DEc) in the mantle of the flat or round valve are consistent with the DEGs determined in the transcriptome analysis (**Supplementary Fig 20**). The subcellular distribution of candidate lncRNAs in mantle cells was explored using qRT-PCR. Expression of *SMPDR* and *TIMPDR* in the nucleus of flat/round valve mantle cells was significantly higher than expression in the cytoplasm (**Supplementary Fig 14a**).

**DEc subcellular localization annotation**

The SignalP5.0 (http://www.cbs.dtu.dk/services/SignalP/) program was used for detection of signal peptides, the TMHMM v.2.0 (http://www.cbs.dtu.dk/services/TMHMM/) was used for detection of transmembrane domains and the DeepLoc v1.0 (http://www.cbs.dtu.dk/services/DeepLoc/) was used for prediction of subcellular localisation. DEc were classified as membrane-spanning if at least one transmembrane region was predicted.

**ProminTools for identification of protein motifs**

DEc were run in the ProminTools package (11) to identify protein motifs in the CyVerse Discovery Environment with default parameters (12) using the *M. gigas* shell proteome as the background. Protein Motif Finder was used to cluster protein motifs on a heatmap and to identify all potential enriched motifs (word clouds) and the Properties Analyzer programme was used to determine the percentage of low protein complexity regions, disordered regions, and the protein charge. To confirm that proteins within each specific cluster were candidate shell matrix proteins (SMPs) they were individually removed, and enrichment analysis was rerun. Differences between foreground (transcriptome) and background (proteome) were automatically generated with the significance set at p > 0.05.

**Non-protein coding gene transcript analysis**

The identity of the non-protein coding gene transcripts identified was reconfirmed considering length (≥ 200 bp) and non-coding potential (13, 14). LncRNAs were distinguished from messenger RNA, sequences using the predictor of lncRNAs and mRNAs based on an improved k-mer scheme (PLEK, (15)) tool and searches against Coding Potential Calculator (CPC, (16)) and Coding-Non-Coding Index (CNCI, (17)) software. Further confirmation of “coding/ non-coding” potential was established by searches against Swissprot (https://www.uniprot.org/) and Pfam (<https://pfam.xfam.org/>).

**Species orthogroup inference**

Protein datasets from the genomes of six bivalve species (**Supplementary Table 1**) were used for species phylogenetic tree analysis. Alternative transcripts were removed from the initial protein data set obtained for each species. Global orthogroup resolution was performed using Orthofinder (ver 2.5.4, parameters -M msa; -T raxml) (18). The resulting tree was displayed in FigTree 1.4.3 (http://tree.bio.ed.ac.uk/software/figtree), rooted with *M. galloprovincialis* and edited in the Inkscape program (https://inkscape.org).

**Optimization of siRNA experimental conditions**

siRNA for candidate lncRNA (*TIMPDR* and *SMPDR*) knock out was tested and optimized using *ex-vivo* cultures of *M. gigas* mantle before advancing for the *in vivo* studies. The factors optimized were the chain (A, B, C, see below), dosage, and duration of exposure to establish the most efficient experimental approach for gene silencing *in vivo*.

***M. gigas ex-vivo mantle cultures***

*Ex-vivo* *M. gigas* mantle cultures were established and optimized for the flat and round valves of 6 adult *M. gigas*. Tissue was extracted from approximately the same position in the mantle from each individual specimen, washed twice in sterile seawater and once in culture medium [30% L15 medium (Sigma-Aldrich), 70% natural filtered sterile seawater (0.22 mm) and 2% FBS (Sigma-Aldrich), pH 8.1 - 8.2) supplemented with a 0.1% penicillin: streptomycin mix (10.000 U:10 mg/ml, Sigma-Aldrich) and 250 μg/ml sterile filtered 1:100 amphotericin B solution (Sigma-Aldrich)]. Tissue samples were cut into approximately 1 mm fragments and the flat mantle from several animals (n = 10) was mixed and randomly assigned to different wells of a 96 well tissue culture plate; the same procedure was applied to the mantle tissue from the round valve. Each well of the tissue culture plate contained approximately 20 mantle fragments and tissues were incubated at 18 °C. Before proceeding to siRNA experiments, expression of candidate genes was confirmed and samples (n = 3) from flat and round valve mantles were collected at 0, 8, and 24 h. The mantle fragments from the cultures were collected by centrifugation at 600 × g, 18 °C for 3 min, immediately frozen on dry ice until used for gene expression analysis, which was carried out as described above. The asymmetric expression characteristics of the five candidate genes in mantle tissue during *in vitro* cultures was maintained for at least 24 hours after establishment of the cultures (**Supplementary Fig 14b**).

***siRNA experimental conditions***

For siRNA studies three siRNA (A, B, C) chains for the target *TIMPDR* and *SMPDR* were designed and supplied by GenePharma (Shanghai, China) (**Supplementary Table 8**). Stock solutions of siRNA were prepared in DEPC-treated water and diluted 100-fold in filtered sterile seawater before the experiments. For chain selection a high (4.4 μg /ml) and a low (1.1 μg /ml) dose of each chain (siRNA-*TIMPDR*) was tested using the *ex vivo* mantle cultures and incubating for 8 h at 18°C and revealed that A chain and C chain had a significant (p < 0.05) inhibitory efficiency (**Supplementary Fig 21a**). To determine the most effective siRNA dose three different concentrations (4.4 μg /ml, 3.3 μg /ml, and 2.2 μg /ml) were tested for chains A and C using the method reported in Zhi et al. 2021 (19) and revealed that chains had a significant (p < 0.05) inhibitory effect at a concentration of 4.4 μg/ml (**Supplementary Fig 21b**). For the selection of the most effective incubation time, the optimal silencing dose (4.4 μg /ml) of either A or C chains were tested, and samples were collected at 2, 4 and 6 h after treatment. Both the A and C chain significantly inhibited (p < 0.001) the expression of *TIMPDR* (inhibition efficiency ~ 75%) after 6 h treatment (**Supplementary Fig 21c**). The strategy used to optimize the dose and incubation time for *TIMPDR* was also used to select the optimal conditions for the siRNA-*SMPDR* chain, using the same concentration range of siRNA, and incubation period and sampling times. The optimal dose at which the siRNA-*SMPDR* C chain could significantly inhibit the expression of *SMPDR* was identified (p < 0.05, **Supplementary Fig 21d**). The siRNA chain and most effective conditions for silencing *TIMPDR* were siRNA-A and C chains at a final concentration of 4.4 μg /ml and an incubation period of 6 h; the same conditions were also selected for *SMPDR* using the siRNA-C chain.

Gene silencing efficiency was determined by quantifying by qRT-PCR the expression of *TIMPDR* and *SMPDR* and their putative gene targets (*NTRDCP* and *Unslp6* and *EGF-CADCP*) in the exposed mantle tissue. A negative control siRNA (NC, GenScript) was included in all experiments using the conditions optimized above for the specific siRNA. Three independent experiments on *M. gigas* mantle cells were performed with the optimised siRNA conditions and RNA extractions and gene expression analysis was performed as described above. SiRNA-LncRNA chains with optimized conditions was confirmed to be effective and siRNA-*TIMPDR* (A and C chain) and siRNA*-SMPDR* C chain significantly inhibited the expression of *TIMPDR* and *SMPDR* (p < 0.05), respectively, and significantly modified the expression of the candidate target genes (p < 0.05, **Supplementary Fig 21e,f**).

**Shell damage-repair assay**

To assess the shell growth rate 3 holes (~ 2 mm in diameter) were drilled in the posterior edge of the juvenile *M. gigas* shell taking care to avoid mantle damage (**Supplementary Fig 2**). The 3 holes were drilled (~4 mm distance between them) with a handheld electric drill (DIATOOLS, China) in the flat and round valves of the same individual, and holes were gently irrigated with clean, filtered seawater to remove any shell dust and fragments. Holes were numbered 1 to 3 in each animal, and shell recovery was monitored across 3 days. Hole 3 was nearest the adductor muscle and hole 1 most distant and the same convention was used for the holes in both the flat and round shell. Shell drilled control and LncRNA injected animals were housed in individual tanks and the progression of shell repair in each hole was inspected after 2 days using a stereoscope (Motic, SMZ-171, China) equipped with a digital camera (Visicam 6 Plus, VWR, Portugal). Two independent experiments were performed. To measure repair digital images of each hole drilled in the shells were taken and the area of shell regrowth determined using ImageJ ver 1.52a. The percentage of shell repair in each valve was calculated for each of the drilled holes as follows: % hole repair area = (repaired area/ total hole area) × 100%. Correlation analysis between the repair rate of the two valves and the shell phenotype (flat and round) was displayed using a forest plot (RevMan 5.0 software).

**Scanning electron microscopy (SEM**)

The repaired shell hole was washed with distilled water, air-dried, and was used for imaging. Shell samples were mounted on stubs coated with gold (JEOL JFC1200, JSM Electron Microscopes, Tokyo, Japan), and were observed in a scanning electron microscope (JEOL JSM5200-LV, JSM Electron Microscopes, Tokyo, Japan) with high-energy beam of 25 kV, and images were acquired after 90 seconds exposure using digital software.

**Supplementary Figures**

**Supplementary Figure 1**

**
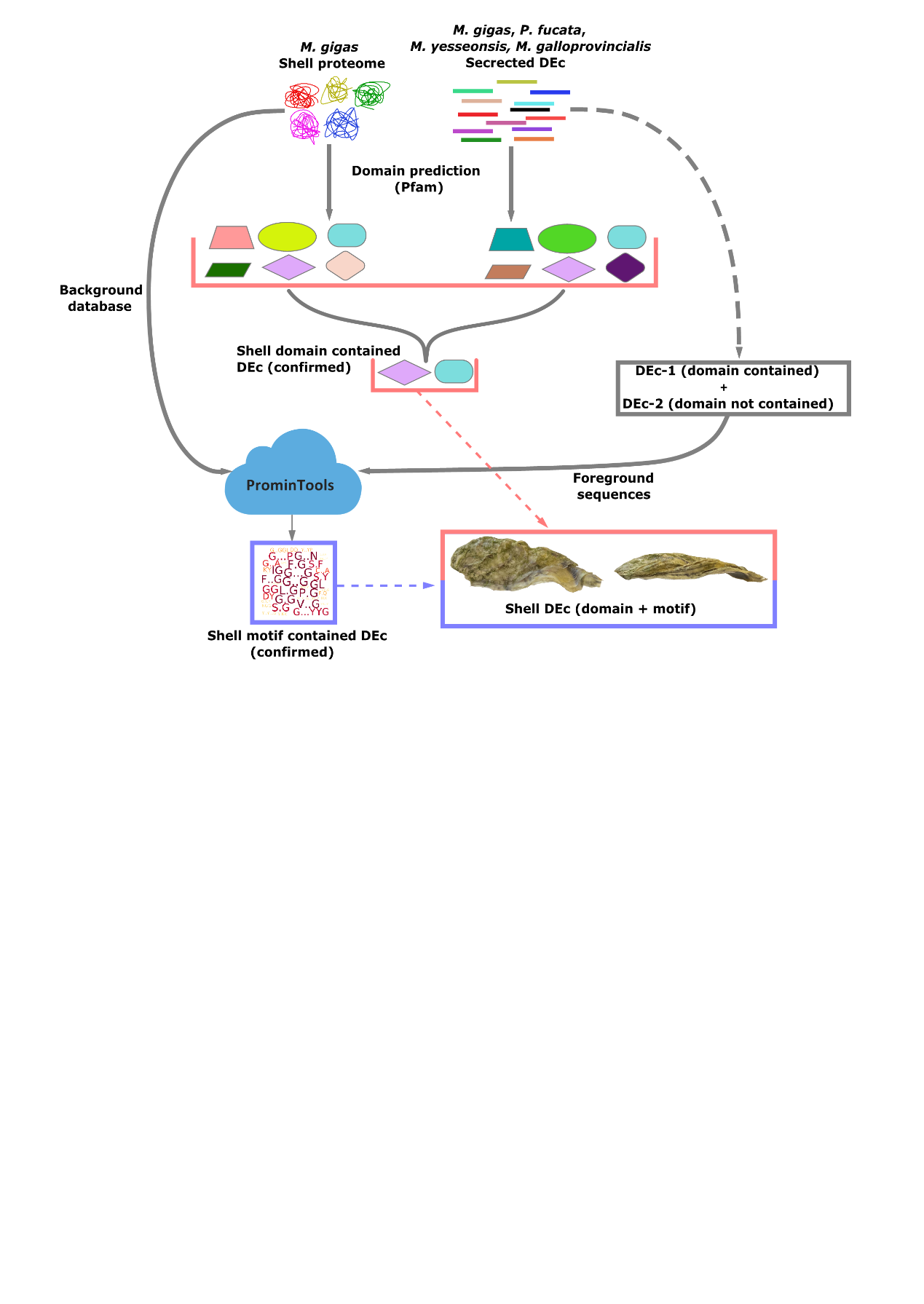
**

**Workflow of the strategy used to characterize the protein domains and motifs of deduced DEc protein genes in the mantle of the asymmetric valves of *M. gigas*, and *M. yessoensis,* and slightly asymmetric valve of *P. fucata* and the symmetric valve of *M. galloprovincialis*.** The DEGs in the mantle transcriptome that code for proteins in *M. gigas* were compared to the shell proteome of *M. gigas* to identify specific protein domains associated with the asymmetric shell. To improve the identification of protein domains associated with bivalve SMPs, DEc genes in the mantle transcriptome that had no significant matches in the species-proteome were subsequently analysed using the ProminTools and employed as foreground groups and compared with the background group (*M. gigas* shell proteome). The dashed line represents DEc genes that had no SMP-domain identified or predict.

**Supplementary Figure 2**

**
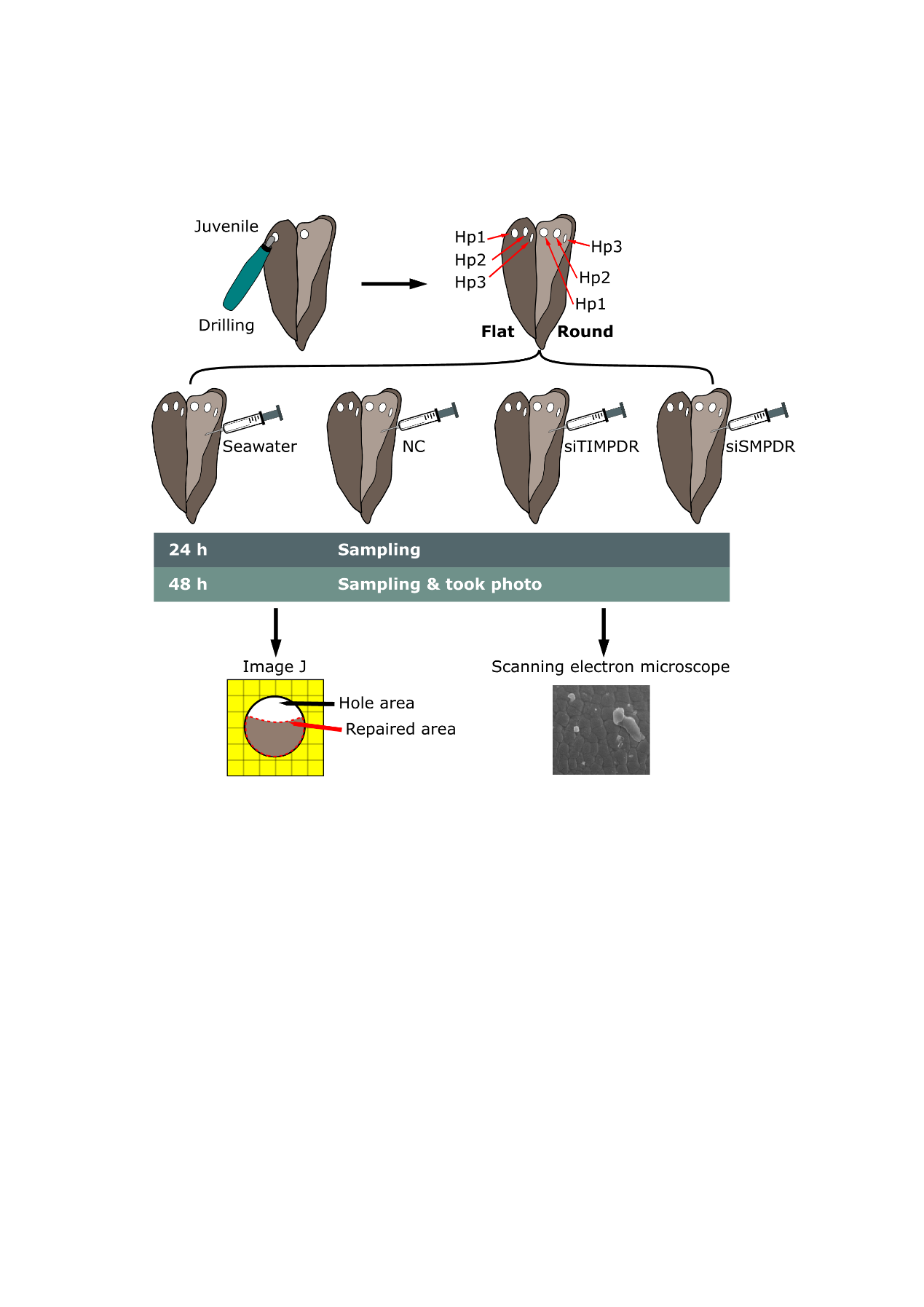
**

**Schematic representation of the strategy taken to test the effect of *SMPDR* and *TIMPDR* on the shell damage-repair process in juvenile *M. gigas*.** Three holes were drilled in each *M. gigas* valve, and the animals were divided into four experimental groups (n = 9 animals/ group – total 54 holes/group): two control (seawater and negative siRNA control chain) and two treatment groups (siRNA-TIMPDR and siRNA-SMPDR). Mantle samples were collected 24 h and 48h after shell damage and the progress of shell repair assessed by taking images of the drilled holes using a digital camera. The microstructure of the newly grown shell that filled the holes was analysed by SEM.

**Supplementary Figure 3**


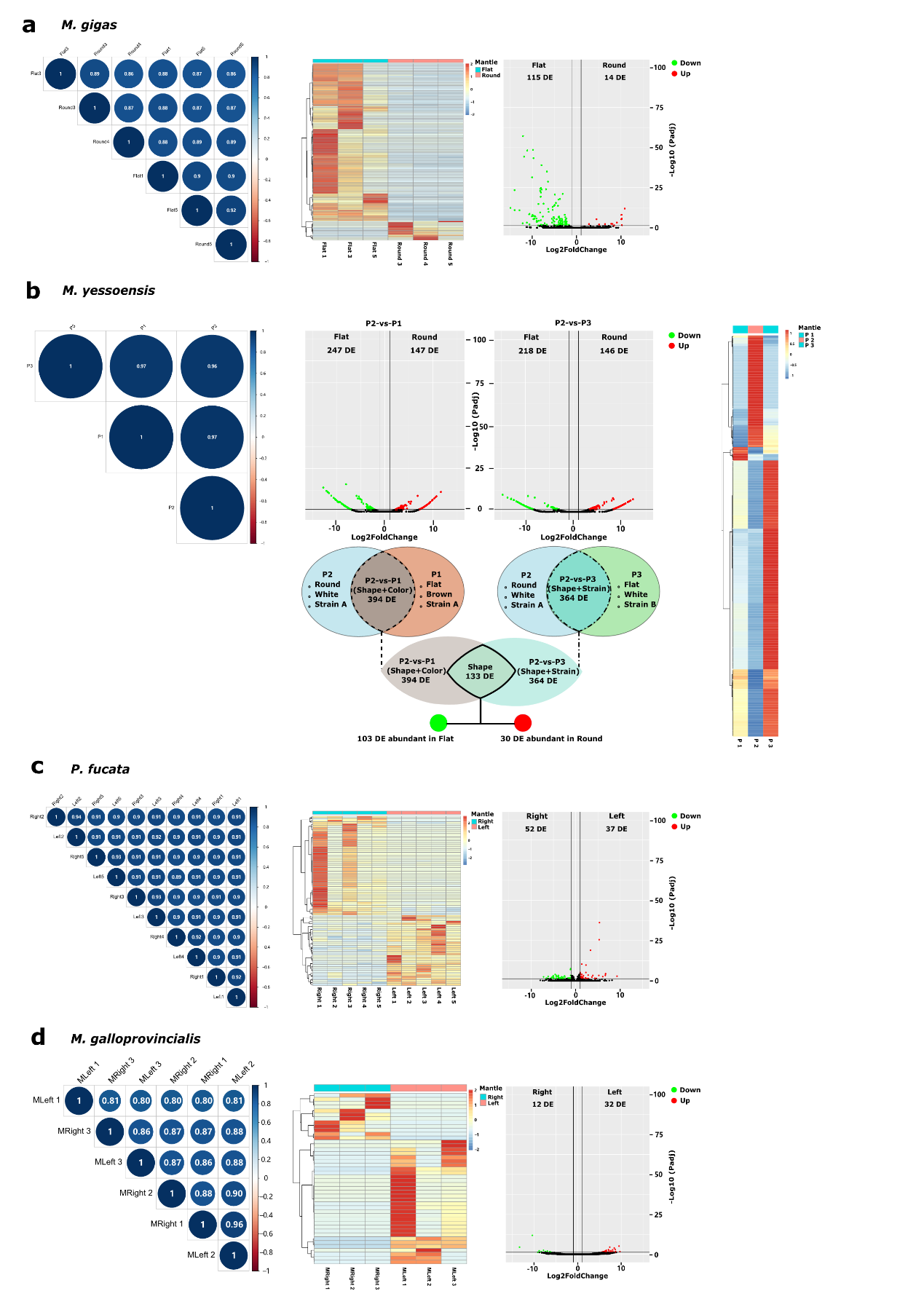


**Correlation analysis of the mantle transcriptome samples and volcano plots and heatmaps of differentially expressed genes. (a, b, c, d)** DEGs in the mantle of two asymmetric bivalves, *M. gigas* **(a)** and *M. yessoensis* **(b)**, in the slightly asymmetric bivalve, *P. fucata* (**c**) and in the symmetric bivalve, *M. galloprovincialis* (**d**). Volcano plots were established with the transcriptome data and statistical significance (log10 adjusted p-value) versus the magnitude of change (log2 fold change) is represented. Heatmaps of the DEG that passed the threshold criteria, a Padj value < 0.05 and log2 fold change ≥ 1, were considered to be significantly modified. The green and red dots correspond to DEGs significantly different in the flat (right) and round (left) valves, respectively. Black dots represent genes that were not significantly DE. The volcano plots were established using the ggplot2 package and the heatmaps using the pheatmap package in R Studio (see Materials and methods).

**Supplementary Figure 4**

**
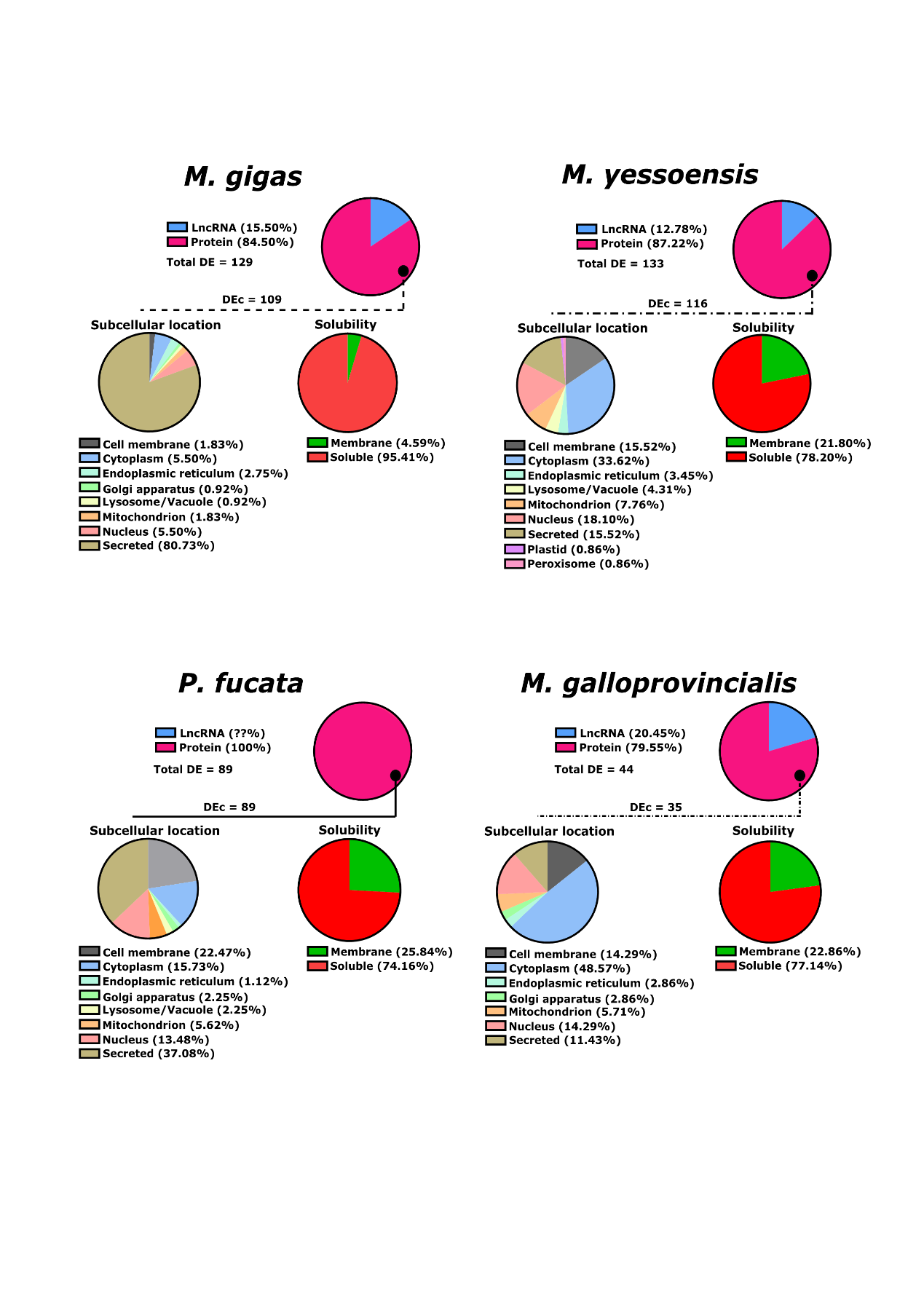
**

**Characterization of the differentially expressed protein coding genes (DEc) in the mantle and their subcellular localization.** Subcellular localization and solubility of DEc genes were predicted using DeepLoc-1.0 (see **Supplementary Table 9**). The majority of the predicted DEc genes are soluble but subcellular localization was variable across the four species.

**Supplementary Figure 5**

**
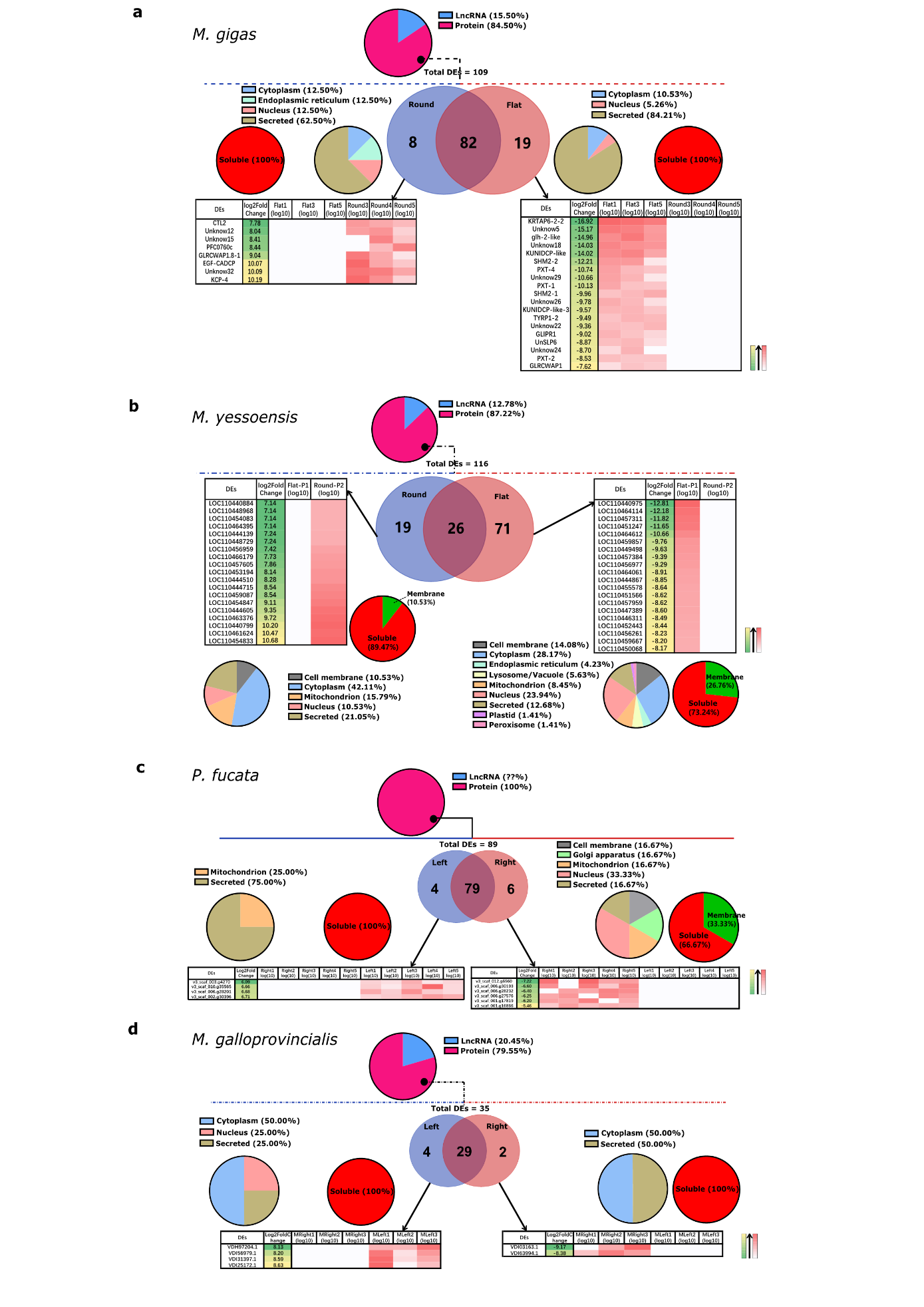
**

**Characterization of specific and common differentially expressed genes (DEG) in the mantle transcriptomes of the flat and round valve. a-d)** Analysis of *M. gigas*, *M. yessoensis*, *P. fucata* and *M. galloprovincialis* mantle transcriptomes. The *M. gigas* and *M. galloprovincialis* mantle transcriptomes were generated in the present study. The *P. fucata* and *M. yessoensis* mantle transcriptomes which are publicly available were downloaded from the SRA database (**Supplementary Table 1**). The Venn diagrams show the number of specific and shared DEG transcripts in the mantle of the flat or round shell. The percentage of DEc genes and lncRNA genes is presented in the pie chart at the top of the figure. DEc genes specific to the mantle of the flat (right) or round (left) valves are indicated in the table and their abundance (heatmap) and subcellular localization is provided.

**Supplementary Figure 6**

**
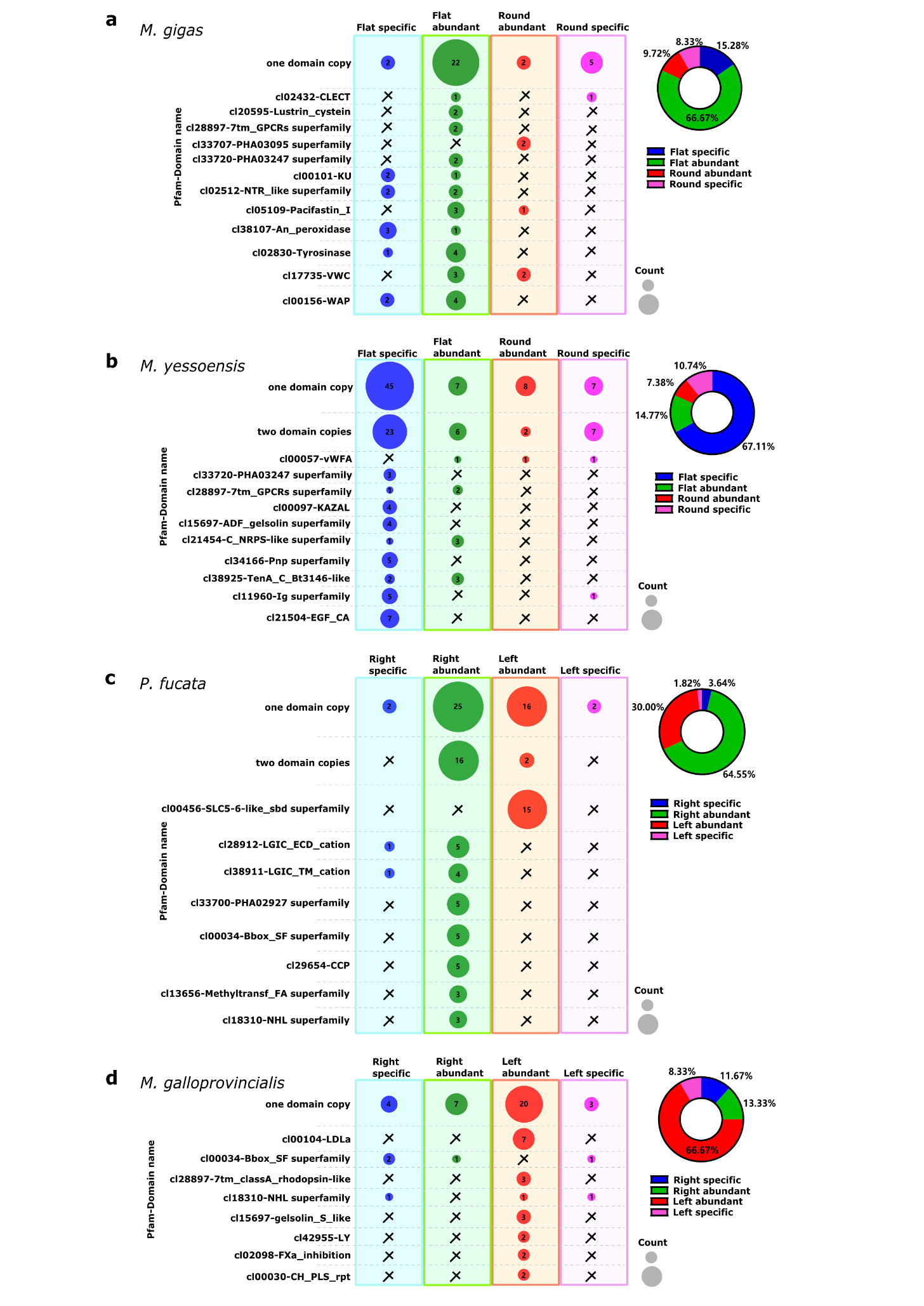
**

**Domain characterization of DE protein coding genes. (a-d)** Analysis of *M. gigas*, *M. yessoensis*, *P. fucata* and *M. galloprovincialis* mantle transcriptomes. Domain characterization of DEc genes was based on Pfam identification (see **Supplementary Table 10**). The protein domains found, and their IDs are provided, and their relative abundance (domain counts) is indicated by the bubble plots. Domains that scored once were grouped in the “one domain” category. For *M. yessoensis* due to the large number of domains found, domains that scored twice in the mantle were also grouped into a specific category, “two domains”. The crosses represent domain absence, and the bubble size is proportional to the number of domains found and the numbers inside the bubbles indicate the domain count. Different colours represent the expression specificity and abundance of DEc genes in the flat and round mantle transcriptomes and the overall percentage of DE domains identified is indicated in the pie chart on the right.

**Supplementary Figure 7**

**
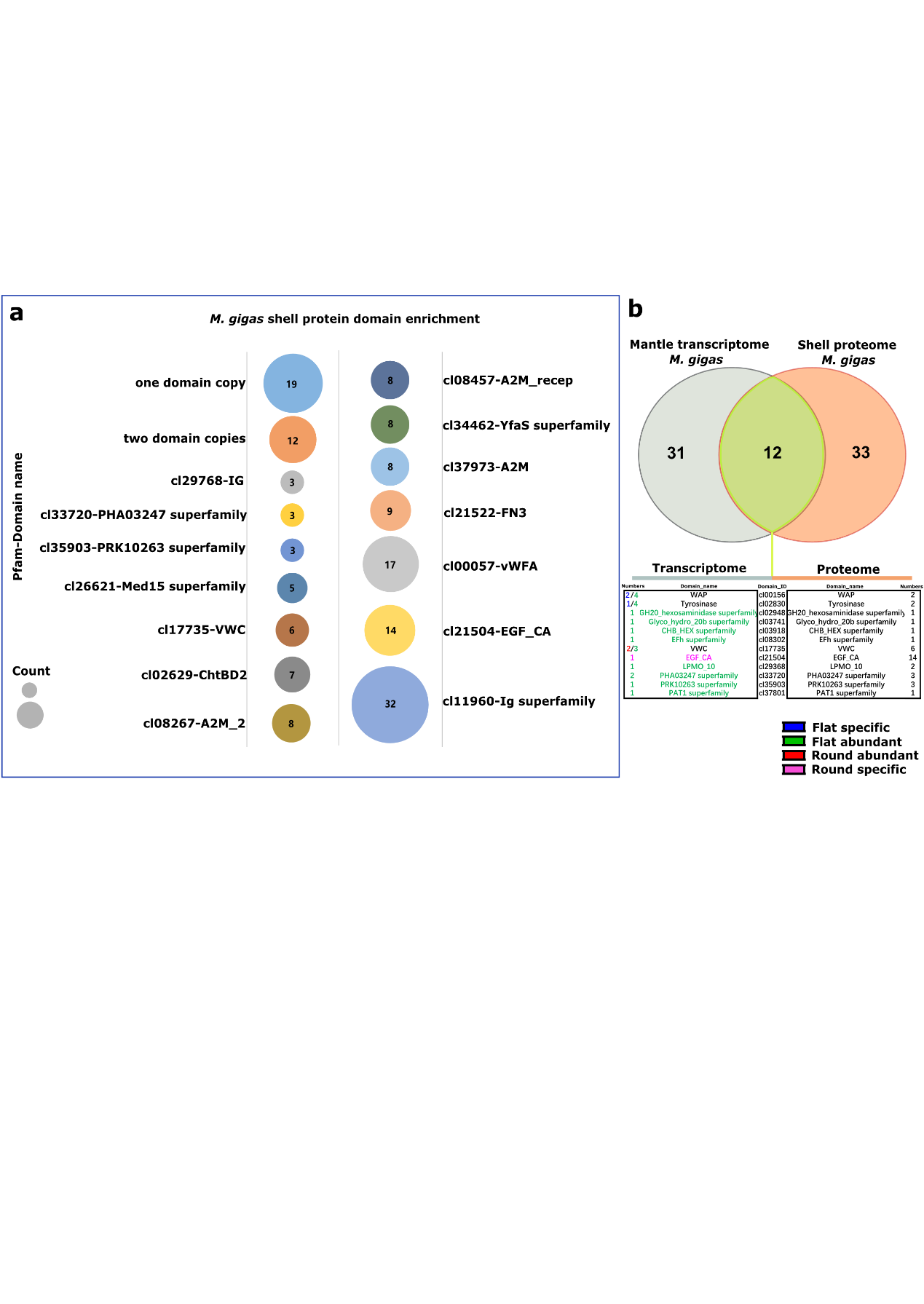
**

**Shell Matrix Protein (SMP) domains in DE protein coding genes in *M. gigas*. (a)** Bubble plots representing the protein domains identified in the *M. gigas* shell proteome (data obtained from Arivalagan et al. 2017) (see **Supplementary Table 11**). Domains that scored once were grouped into the “one domain” category and domains that scored twice were grouped into the “two domains” category. The bubble size is proportional to the number of domains found, and the count number is indicated. (**b**) A Venn diagram showing the specific and shared domains in DEc genes of the mantle transcriptome and the shell proteome (see **Supplementary Table 12**). The table presents 12-shared protein domains and their ID codes and the number of domain counts present in the DEc genes in the *M. gigas* mantle transcriptome and proteome. The colour code represents their expression and abundance in the mantle of the flat and the round valves.

**Supplementary Figure 8**

**
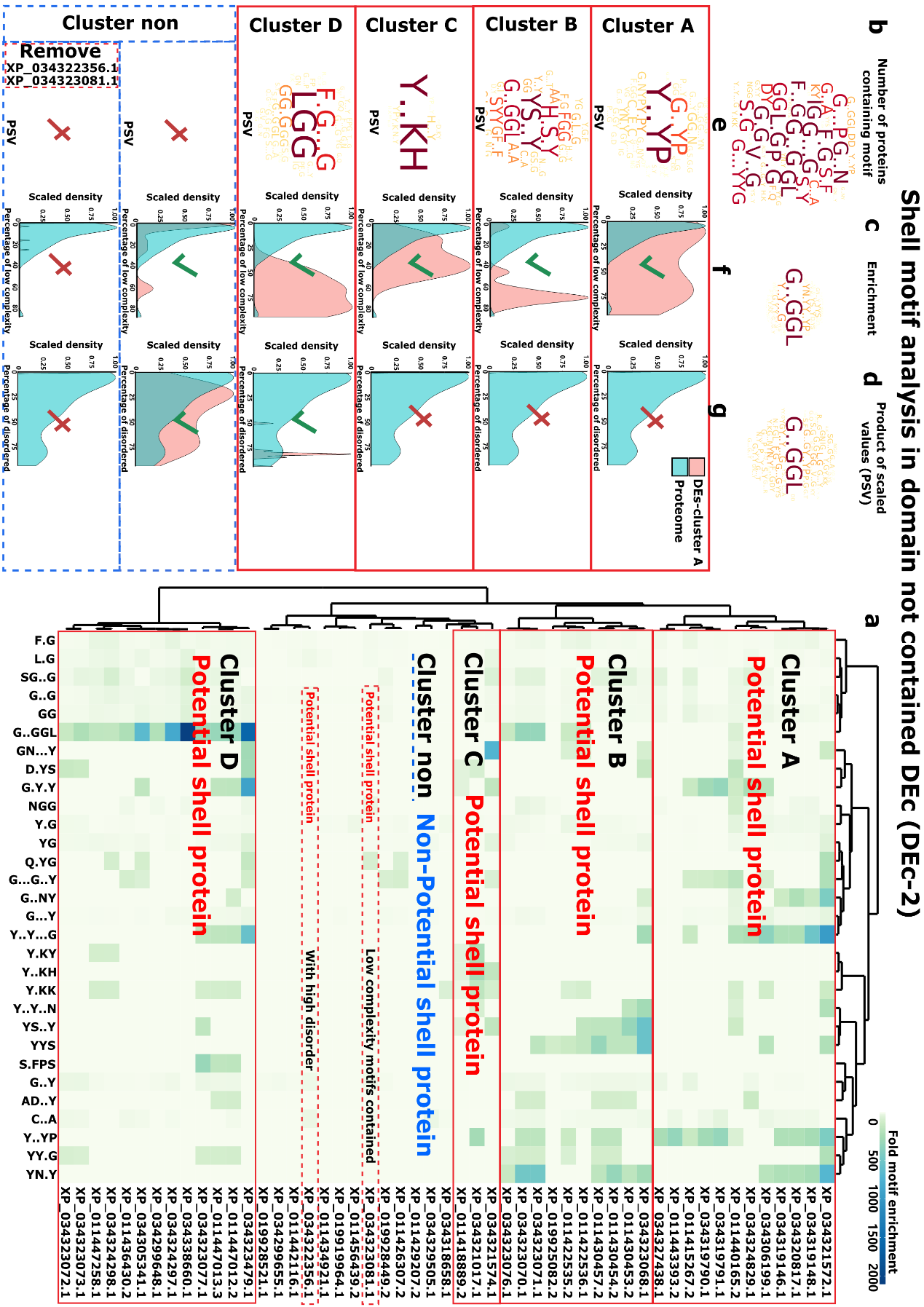
**

**Identification of putative SMP-motifs in non-annotated DE protein coding genes in the mantle transcriptome using ProminTools. (a)** A heatmap showing the clustering of the predicted proteins (DE protein coding genes) based on motif enrichment analysis. Protein clustering was restricted to the 30 motifs with the greatest overall enrichment and according to the foreground knowledge proteins were classified within clusters A to D, and proteins that were not enriched where classified as non-SMP. Proteins lacking enriched motifs were found but are not displayed in the figure. (**b)** predicted motifs and (**c**, **d)** enriched motifs present in at least 5% of all the predicted DEc genes in the mantle relative to the background established using the *M. gigas* shell proteome (Arivalagan et al. 2017). Font size in the word cloud is proportional to the overall motif abundance. Analysis of the deduced individual protein clusters according to enriched protein motifs in each protein cluster, (**f)** percentage of low complexity sequences (i.e, single amino acids repeats or short amino acid motifs) and (**g)** percentage of disordered sequence structure. The area below the curves corresponds to the distribution of the low complexity sequences in the proteins common to the foreground and background sequences. DE genes are in pink, and the shell proteome is in blue. Putative SMPs (see **Supplementary Table 12**) were designated when they met two of the criteria listed in e - g and are identified with √.

**Supplementary Figure 9**

**
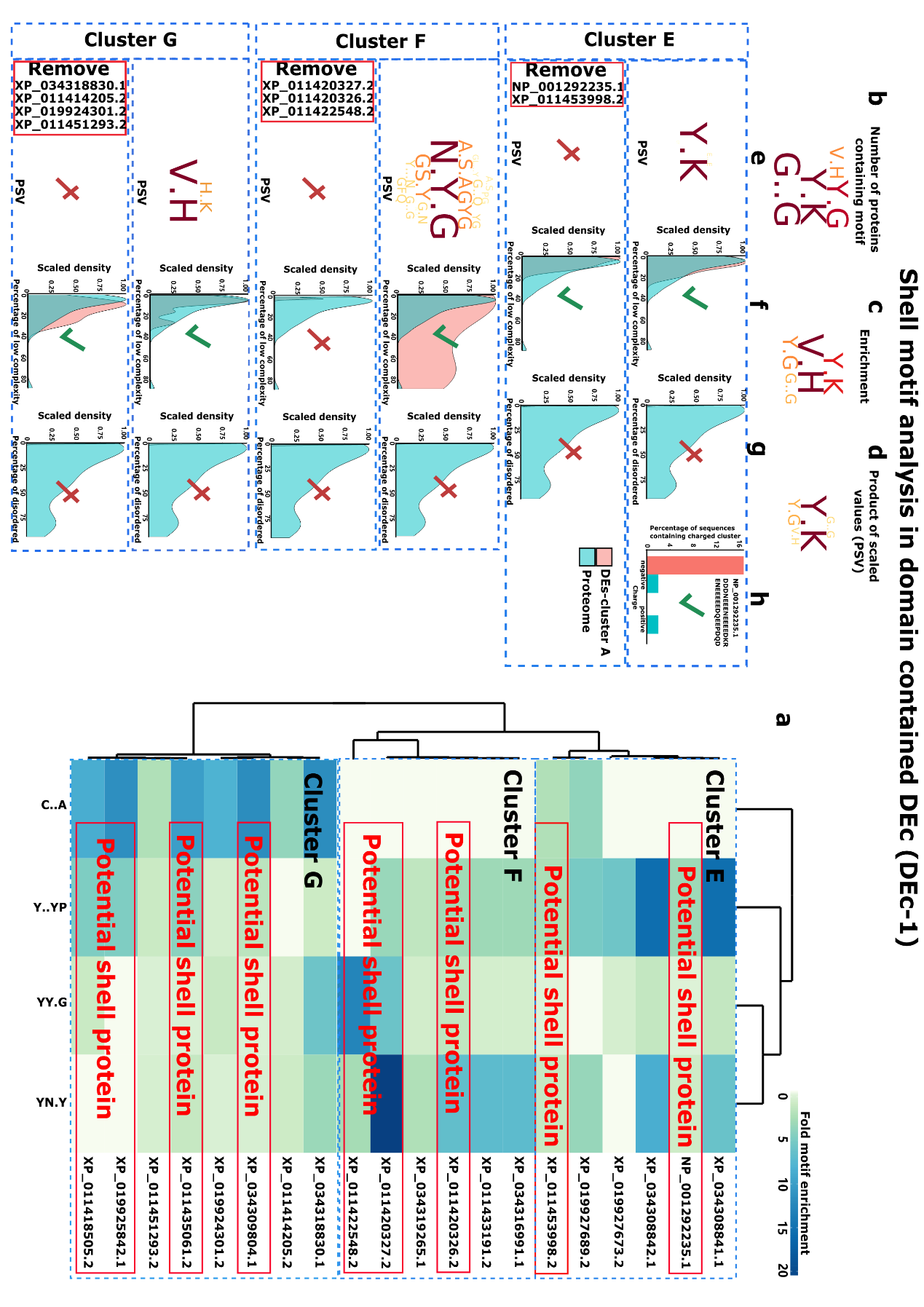
 Identification of SMP-motifs in annotated DE protein coding genes in the mantle using the ProminTools. (a)** A heatmap showing the clustering of the predicted proteins based on motif enrichment analysis. Protein clustering was restricted to the 30 motifs with the greatest overall enrichment based on comparisons with the foreground proteins (from proteomics) and were separated between clusters E to G. (**b)** predicted motifs and (**c**, **d)** enriched motifs present in at least 5% of all the predicted proteins of the DEc genes in the mantle relative to the background established using the *M. gigas* shell proteome (Arivalagan et al. 2017). Font size in the word cloud is proportional to the motif abundance. Analysis of the individual protein clusters was based on (**e)** the enriched protein motifs in each protein cluster, (**f)** the percentage of low complexity sequences (i.e, single amino acid repeats or short amino acid motifs) (**g)** the percentage of disordered sequence structure and (**h)** the percentage of sequences containing charged clusters. The area below the curves corresponds to the distribution of the low complexity sequences in the proteins common to the foreground and background sequences. Deduced DE protein coding genes are in pink, and proteins of the SMP are in blue. DEc genes were designated as a putative SMPs (see **Supplementary Table 12**) when they met two of the criteria listed in e - h and are identified with √.

**Supplementary Figure 10**

**
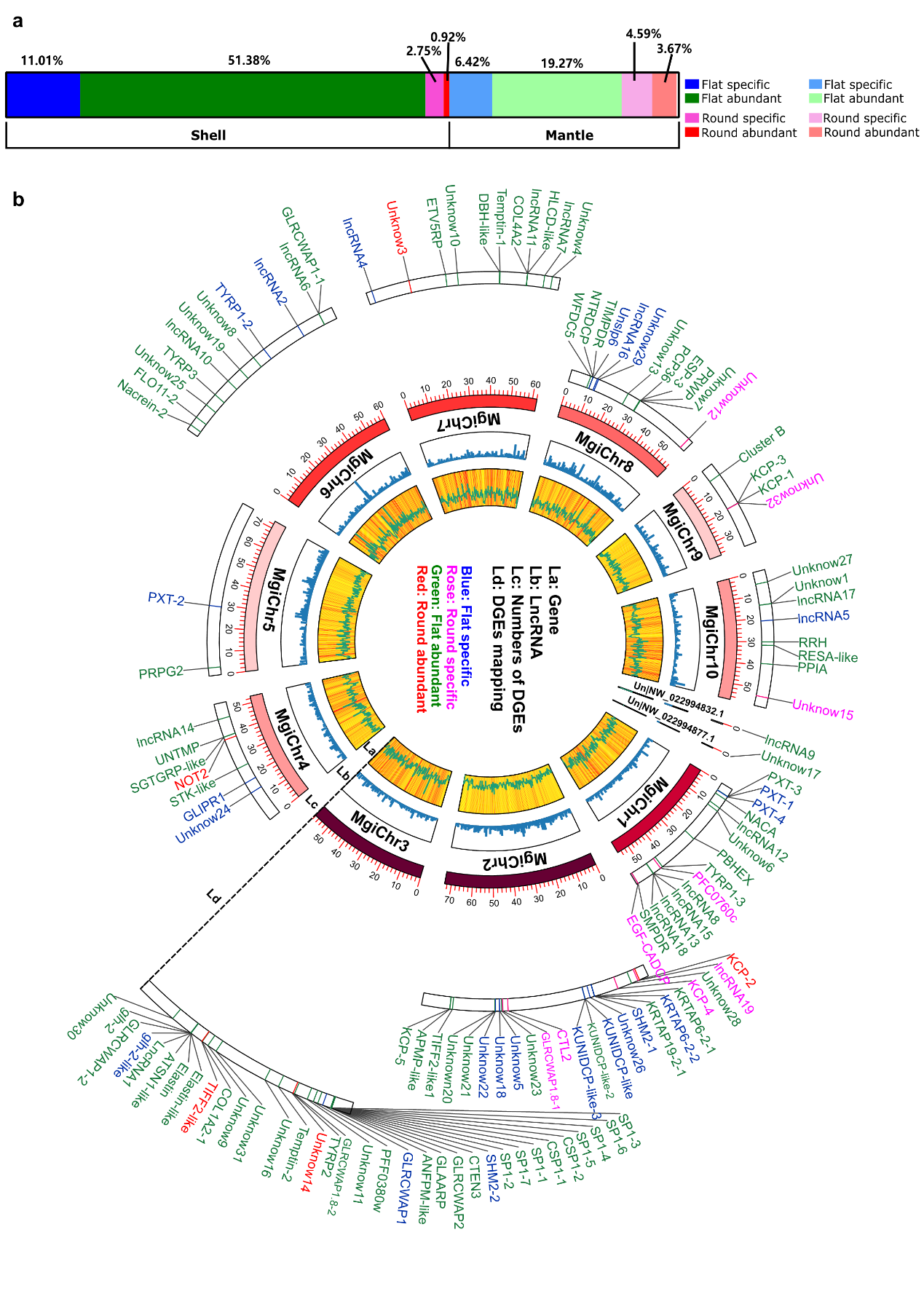
**

**The relative abundance of DEc genes between the mantle of the flat and round valve and their position in the genome**. **(a)** Overall description and relative abundance of the DEc genes in the *M. gigas* mantle. The percentage of transcripts and their relative abundance were grouped into putative SMPs or other non-SMP proteins of the mantle. Most DEc genes are related to SMPs and abundant or specific to the *M. gigas* flat valve mantle. **(b)** Genome mapping of the DE transcripts (protein coding and lncRNAs) identified between the mantle of the flat and round valves in *M. gigas*. The interior circle (La) represents the mapping of all genes, the outer circle (Lb) the mapping of the long non-coding (lncRNA) genes and the red bars (Lc) above the chromosomes represent the mapping of the mantle DEGs with the colour intensity indicating their relative abundance. A large percentage of the mantle DEGs mapped to *M. gigas* chromosome 2 and 3 (dark red). The relative position (Ld) of all the DEGs from the mantle transcriptome comparison within each chromosome is presented and gene names are coloured according to their specificity and abundance in the mantle of the flat and round valves.

**Supplementary Figure 11**

**
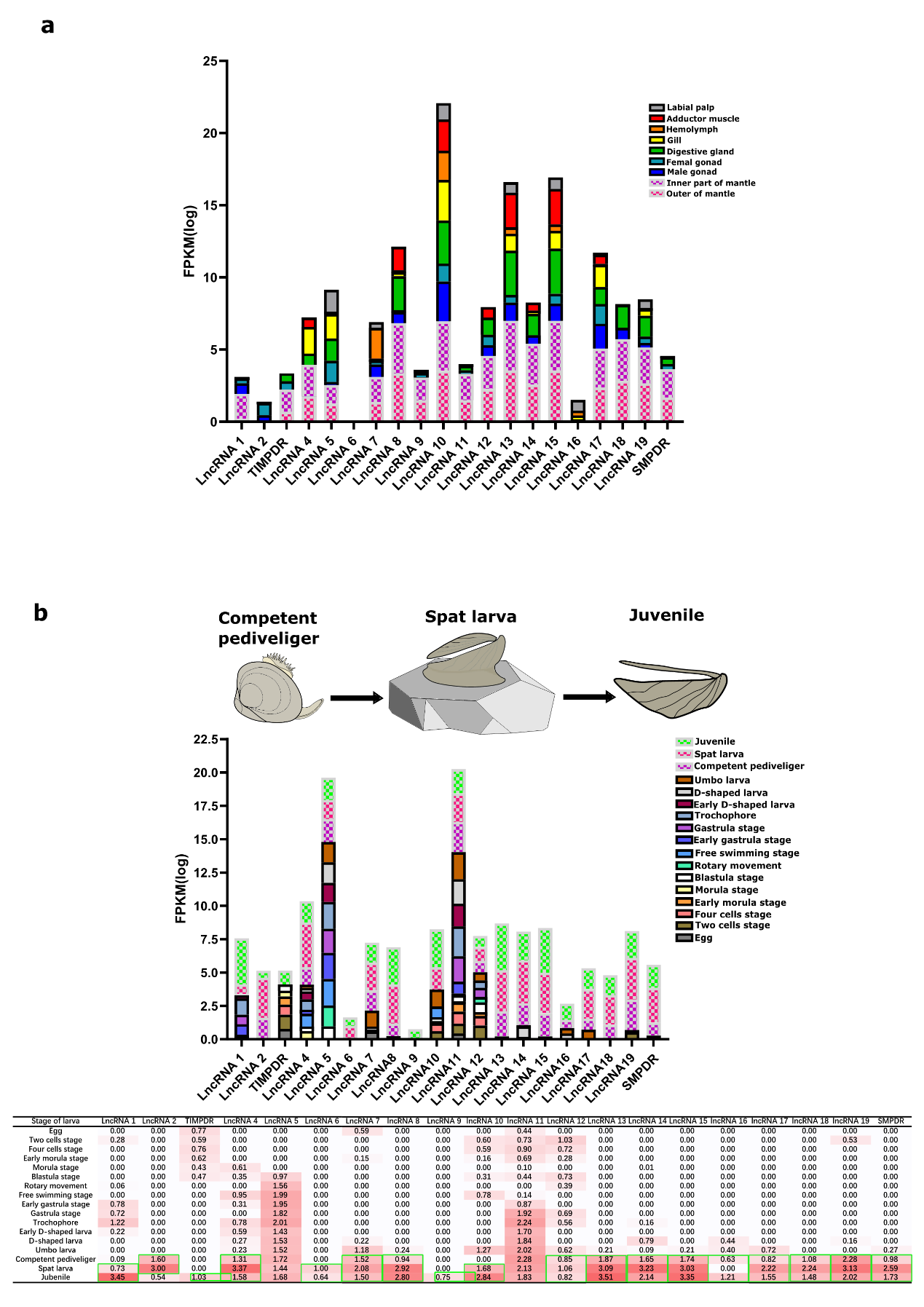
**

**Expression and distribution profile of the selected DE lncRNAs (lncRNAs 1 to 20) in the *M. gigas* mantle of the flat and round valve.** Transcriptome data was obtained from public databases and the SRA accession numbers are provided in **Supplementary Table 1** and transcript expression is given in logFPKMs. (**a)** The expression and abundance in adult *M. gigas* tissues. The colours represent the different tissues analysed. (**b**) Expression and abundance in different stages of developing *M. gigas* larvae. The coloured bars represent different larval stages. Transcripts with a significantly modified expression in larval stages associated with the establishment of the shell and its shape (competent pediveliger, spat larva and juvenile) compared to other stages were considered as candidates for further studies. The heatmap represents relative transcript abundance in different developmental stages and the candidate transcripts selected are boxed in green.

**Supplementary Figure 12**


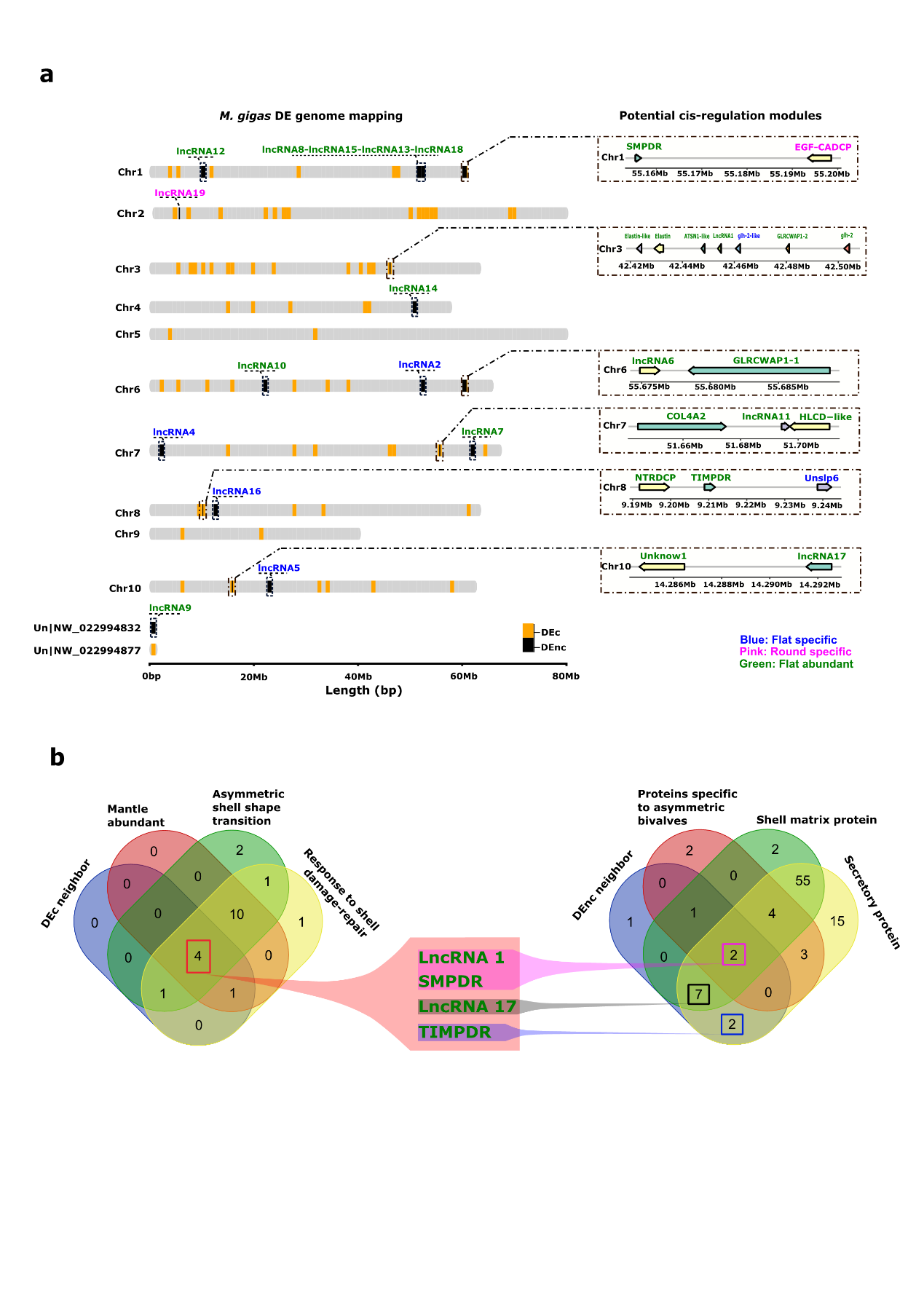


**Mantle lncRNAs and candidate cis-regulatory modules in *M. gigas*.** (a) Mapping of DEGs in the *M. gigas* genome and identification of potential cis-regulation modules were based on the distance (kb) between protein-coding and lncRNA genes in the genome (located < 100 kb distant). DEc genes are represented by yellow bars and lncRNA genes by back bars. Six DEG candidates with transcriptional regulatory activity (cis-regulatory modules) were identified. Arrows indicate gene orientation and gene position is provided (Megabases, Mb). Protein coding gene symbols and lncRNAs are indicated and are coloured according to distribution and abundance in the *M. gigas* mantle. **(b)** The criteria for the identification of candidate lncRNAs putatively assigned as cis-regulatory factors was based on their expression profile and position in the genome. The Venn diagram shows the number and grouping of lncRNAs by 1) regulated response to shell damage-repair (yellow), 2) regulation in larval stages associated with shell shape transition (green), 3) expression abundance in the mantle (red) and 4) proximity with a DEc gene (< 100 kb distance) (blue). LncRNA1, lncRNA17, *TIMPDR* (TIMP Domain Regulator) and *SMPDR* (Shell Matrix Protein-Domain Regulator) were identified across the 4 analysed conditions and were abundant in the mantle of the *M. gigas* flat valve (green). Characterisation of DEc genes potentially regulated by the candidate lncRNAs. The Venn diagram shows the number and overlap of the DEc genes according to the criteria previously used: 1) secretory protein (yellow), 2) putative SMP (green), 3) proteins with domains specific to the asymmetric bivalve mantle (red) and 4) localized in the neighbourhood (< 100 kb distance) of a DE lncRNA (blue). *EGF-CADCP* (EGF-CA domain containing protein) (under potential regulation of *SMPDR*) is specific to the mantle of the round valve and ATSN1-like (under potential regulation of lncRNA1) is abundant in the mantle of the flat valve and both are likely to be secretory SMPs, and common in the mantle of bivalves with an asymmetric shell. The *NTRDCP* gene is abundant in the mantle of the flat valve and *Unslp6* gene is a mantle specific gene associated with the flat shell, and both are potentially under the regulation of *TIMPDR* and are likely to encode putative secretory proteins.

**Supplementary Figure 13**

**
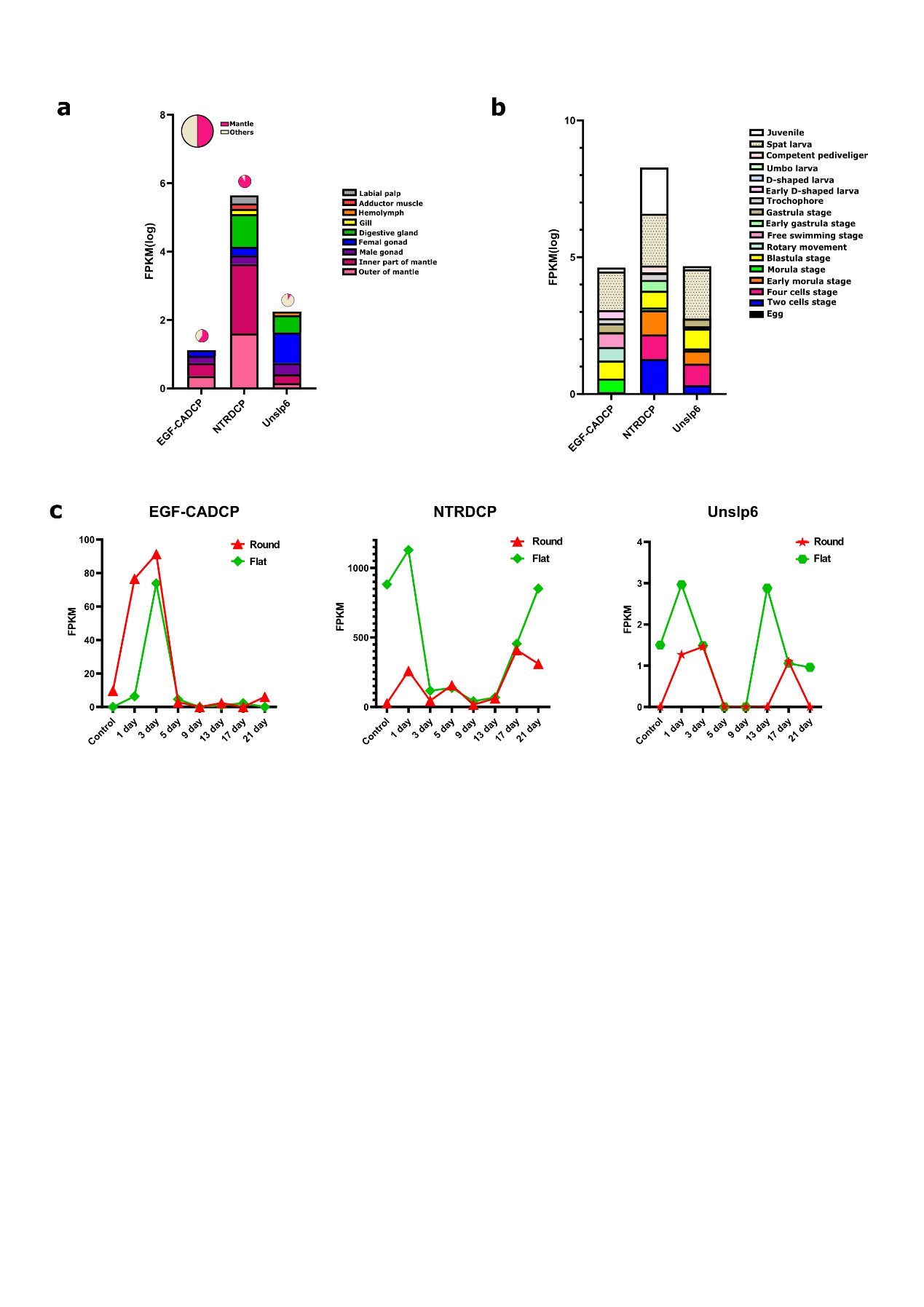
**

**Expression and profile of the tissue distribution of the candidate protein coding genes regulated by lncRNA.** The candidate protein coding genes are *EGF-CADCP*, *NTRDCP* and *Unslp6*. Transcriptome data of the mantle from the flat and round valves was obtained from publicly available data and their SRA accession numbers are available in **Supplementary Table 1**. Transcript expression is given as logFPKM (**a**) Transcript expression and abundance in adult *M. gigas* tissues. The colours represent the different tissues and the pie chart at the top of each bar represents the relative representation of transcripts in the mantle compared to other tissues. (**b**) Transcript expression and abundance in different *M. gigas* developmental stages. The coloured bars represent different larval stages. (**c**) Transcript expression during shell damage-repair (after 21 days recovery) in adult *M. gigas*. Transcripts in the mantle from the flat or round valve are represented by green and red lines, respectively.

**Supplementary Figure 14**

**
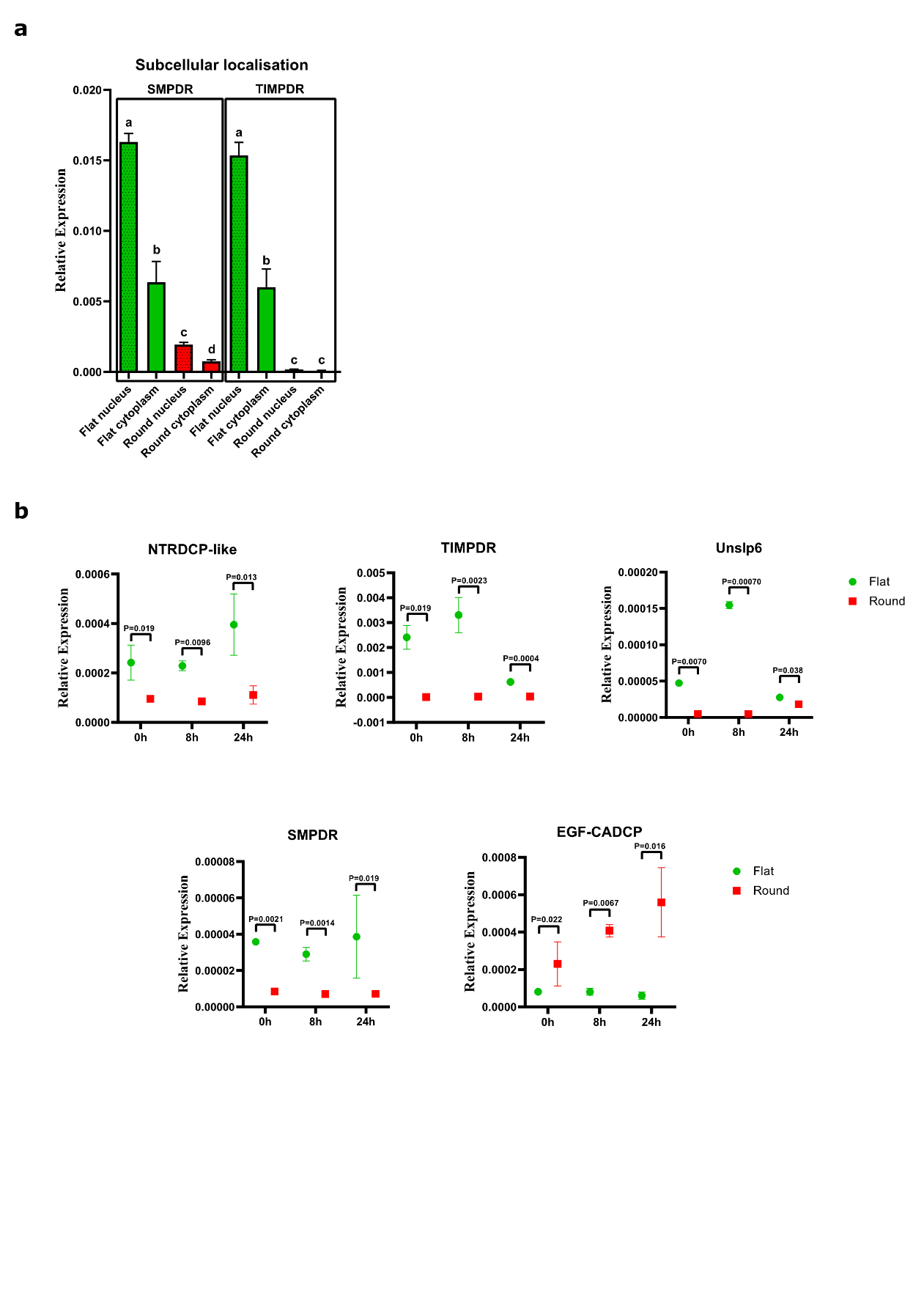
**

**Expression of lncRNA and protein coding candidate genes in the *M. gigas* mantle.** (**a)** Subcellular localization (nucleus, N or cytoplasm, C) of the target lncRNA transcripts (*TIMPDR* and *SMPDR*) in *M. gigas* mantle from the flat or round valve. The mantle from the flat (green) and round (red) valve were collected from adult *M. gigas* and both lncRNA transcripts were more abundant in the flat mantle and predominantly found inside the nucleus. The relative gene expression levels were determined by qRT-PCR and calculated as the fold change after normalization by the average expression of the reference genes (EF1α and RL7). Data represents the mean of n = 3 biological replicates (each biological replicate represents a pool of 3 individuals) and is shown as the mean ± SEM. One-way ANOVA was performed to detect significant differences between samples and different letters indicate significant differences (p < 0.05). **(b)** Expression of lncRNA transcripts in the mantle of the flat (green) and round (red) valves of juvenile *M. gigas*. Mantle tissue fragments were collected from an *ex-vivo* tissue culture at different time points (0, 8 and 24h). Gene expression was determined by qRT-PCR and calculated as the fold change after normalization by the averaged expression of the reference genes (*EF1α* and *RL7*). Data is the mean ± SEM of 3 biological replicates (each biological replicate represents a mixture of ± 20 mantle fragments from 6 individuals) and One-way ANOVA was performed to identify significant differences between samples. The p value is shown, and significance was considered at p < 0.05. No differences in the abundance of gene transcripts existed between the samples at 8 hours compared to 0h in the control.

**Supplementary Figure 15**

**
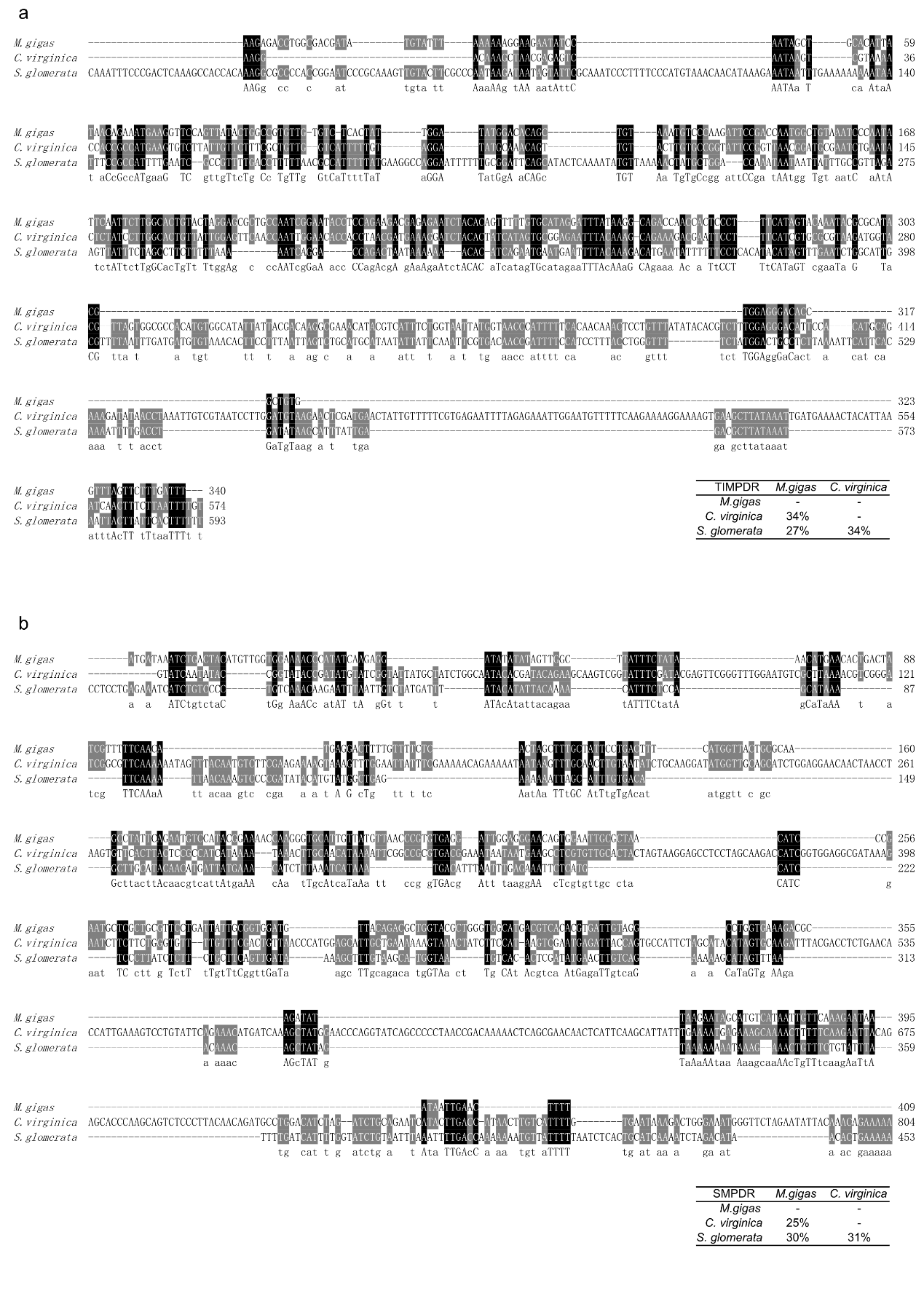
**

**Multiple sequence alignment of the *M. gigas* *TIMPDR* and *SMPDR* and homologues in *C. virginica* and *S. glomerata*.** Multiple sequence alignments of the nucleotide sequence of (**a)** *TIMPDR* and (**b)** *SMPDR* from *M. gigas* and the homologues in *C. virginica* and *S. glomerata*. The *C. virginica* sequences were obtained from the annotation of the genome with predicted gene transcripts (GCA_002022765.4) and from the *S. glomerata* by searching against the SRA data. The sequences retrieved were predicted to be lncRNAs **Supplementary Table 13**. Sequence alignments were performed in Aliview with MUSCLE and the aligned sequences were edited and the percentage of sequence identity calculated in GeneDoc.

**Supplementary Figure 16**

**
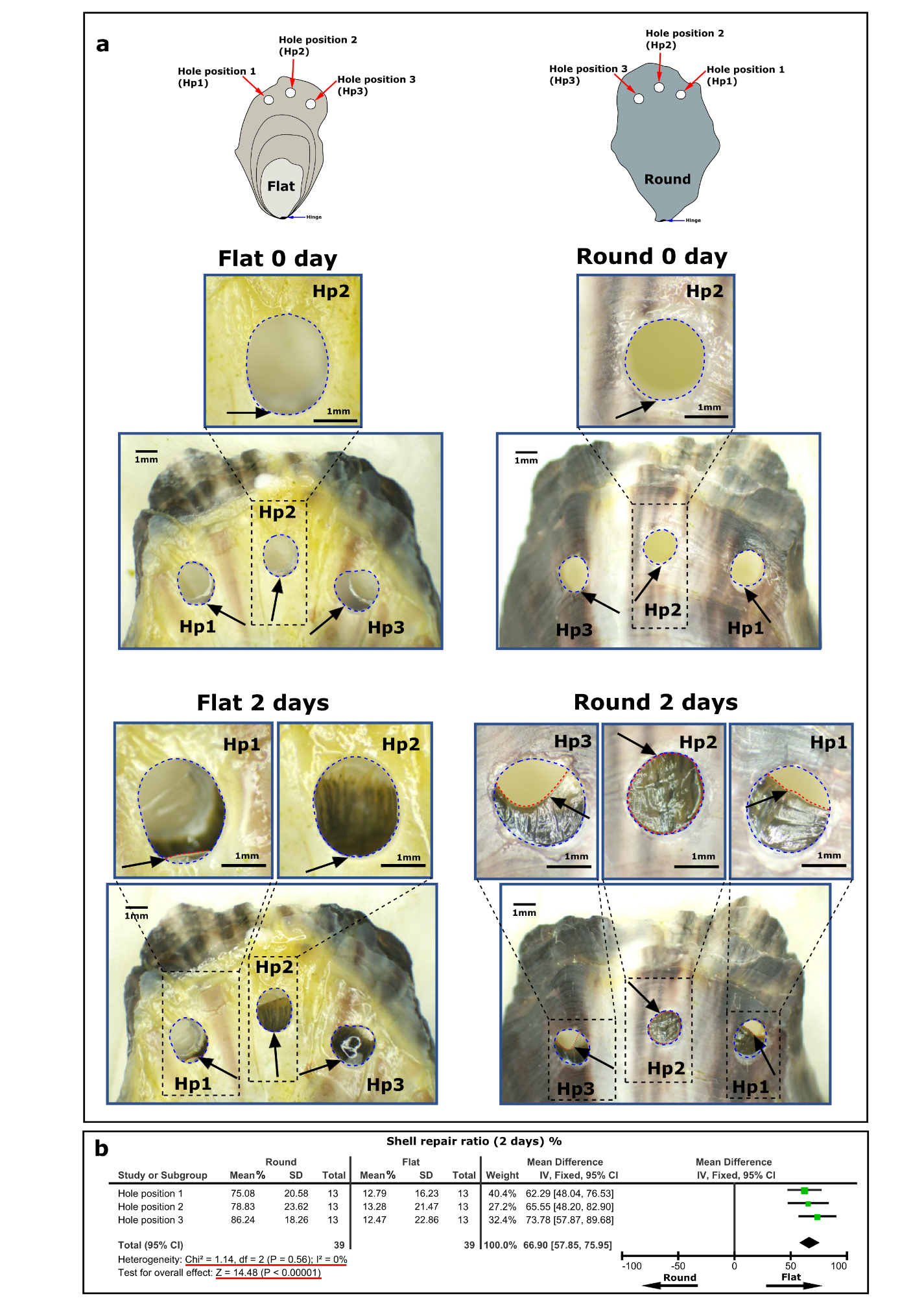
**

**Shell damage-repair assays in juvenile *M. gigas*. (a)** A schematic representation of the shell damage drilling experiment. Three holes (Hp1, Hp2 and Hp3) were drilled at the edge of the shell of each animal using a handheld electric drill (DIATOOLS, China) with a 2 mm metal drill head. The shell was pierced through, and three identical holes were introduced in approximately the same position (Hp) in the flat and round shell of each individual in the experiment. Care was taken to avoid damaging the mantle during drilling. Bright field digital photographs were taken of the holes in the flat and round shells of *M. gigas* at day 0 and day 2 during recovery. Images were taken using a stereoscope (Motic, SMZ-171, China) equipped with a digital camera (Visicam 6 Plus, VWR, Portugal). The arrows in the images indicate the side of the hole that is closer to the shell hinge. For day 0 a magnified image of a typical hole is provided using a selected image of Hp2 in the flat and round side of the shell. For day 2 detailed images of Hp1 and Hp2 are shown for the flat valve and for the round valve Hp1, Hp2 and Hp3 are shown. The red-dashed lines delimit the newly growth shell in each hole. **(b)** The shell damage-repair ratio of the holes punctured in the flat and round valves. Analysis was performed after 2 days shell regrowth and the area of the newly grown shell (repaired shell) in relation to the total hole area n = 13 individuals/3 holes/valve was calculated using ImageJ ver 1.52a software. The mean and standard deviation of the percentage of repair for each hole (Hp1, Hp2 and Hp3) in the flat and round valves are presented in the table. The mean difference in repair for the three holes made in each of the valves was used to draw a forest plot (RevMan 5.0 software). In the forest plot the x-axis represents the shell valves and the different hole position in the shell is plotted as a green box and the line indicates the 95% confidence interval. The diamond at the bottom shows the averaged combined data from all the individuals. Heterogeneity and overall effects are highlighted by the red underline. The scale bar in the photographic images represents 1mm. The statistical analyses presented in the figure were obtained from the software output (RevMan 5.0).

**Supplementary Figure 17**

**
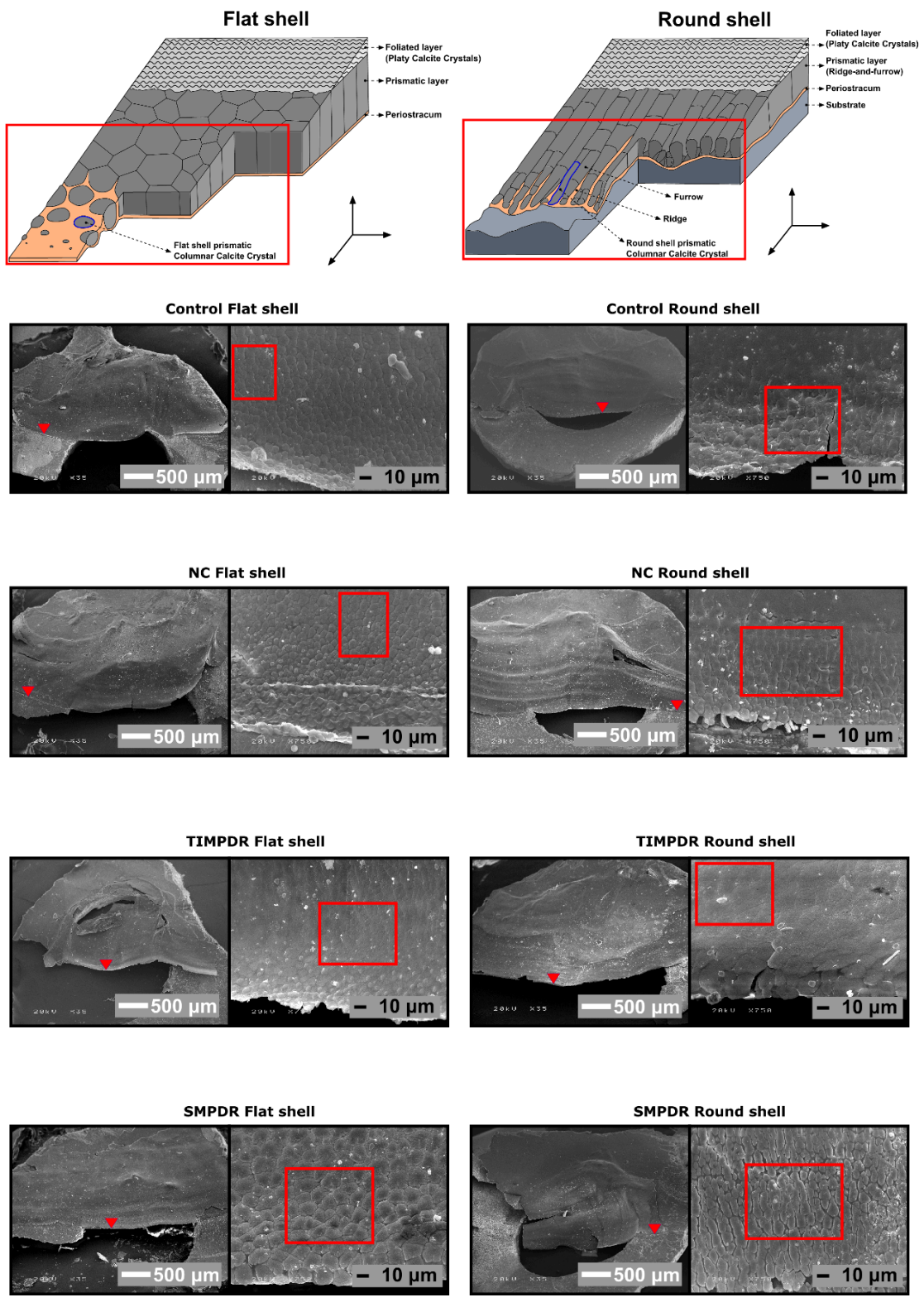
**

**Scanning electron microscopy (SEM) images of the prismatic layer of the recovered shell of the flat and round valves after drilling.** The 3D models at the top of the figure represent the micro structure of the normal flat and round valve near to the edge (modified scheme from (20)). The 3D models were designed using Inkscape ver1.1.2 (The Inkscape Team). Photos (×35) represent a panorama captured for each sample, with red triangles identifying the position boxed in red in the higher magnification image of the captured area. The red rectangles identify the regions that are also shown in **Fig 4d** of the main article.

**Supplementary Figure 18**

**
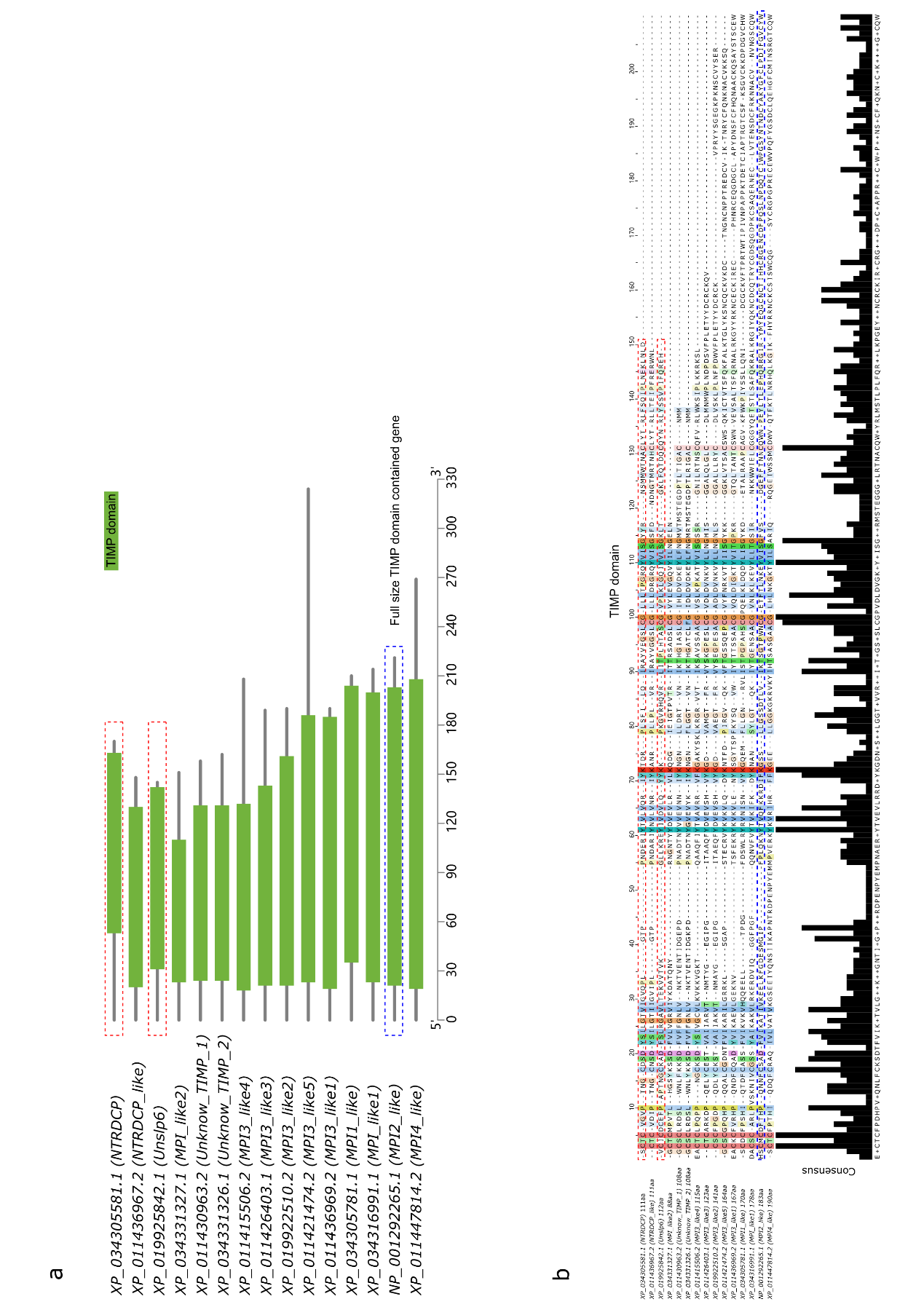
**

**Multiple sequence alignment of the** ***TIMPDR* candidate regulatory proteins and homologue genes containing TIMP domains in the genome of *M. gigas*.** *TIMPDR* candidate regulatory NTRDCP and Unslp6 proteins, both contained one TIMP domain. Three copies of the *NTRDCP* gene occur in the genome, and two are expressed in the mantle, but only one copy of *Unslp6* gene was identified (see **Supplementary Table 11**). In addition, 43 genes in the genome encoded proteins that contain only one TIMP domain, 30 of which are expressed in the mantle (see **Supplementary Table 11**). **(a)** a schematic representation of the multiple sequence alignment of the deduced full-length proteins of genes containing a TIMP domain that are expressed in the mantle. Using the TIMP domain length recorded in Pfam as the alignment standard, 12 TIMP domains of differing lengths were identified. NTRDCP, NTRDCP-like2, Unslp6 and 12 proteins with different TIMP domain lengths were used for the multiple sequence alignment. *TIMPDR* candidate regulatory proteins are within red dashed boxes, and deduced proteins containing the full-length TIMP domain are within blue dashed boxes. **(b)** Multiple sequence alignment of the TIMP domain. Sequence alignments of the retrieved data was performed in Aliview with MUSCLE and the aligned sequences were edited and the consensus sequence obtained in Jalview v2.11.2.3. The identity threshold display of amino acid letters is 30, and the background colour is adopted from ClustalX. *TIMPDR* candidate regulatory proteins are marked within red dashed boxes, the protein containing a full-length TIMP domain is marked with a blue dashed box. The TIMP domain in the *Unslp6* gene is unique. Although the TIMP domain in the *NTRDCP* gene has only limited specificity, its gene expression level in the mantle is higher than that of *NTRDCP-like1* and *NTRDCP-like2*.

**Supplementary Figure 19**

**
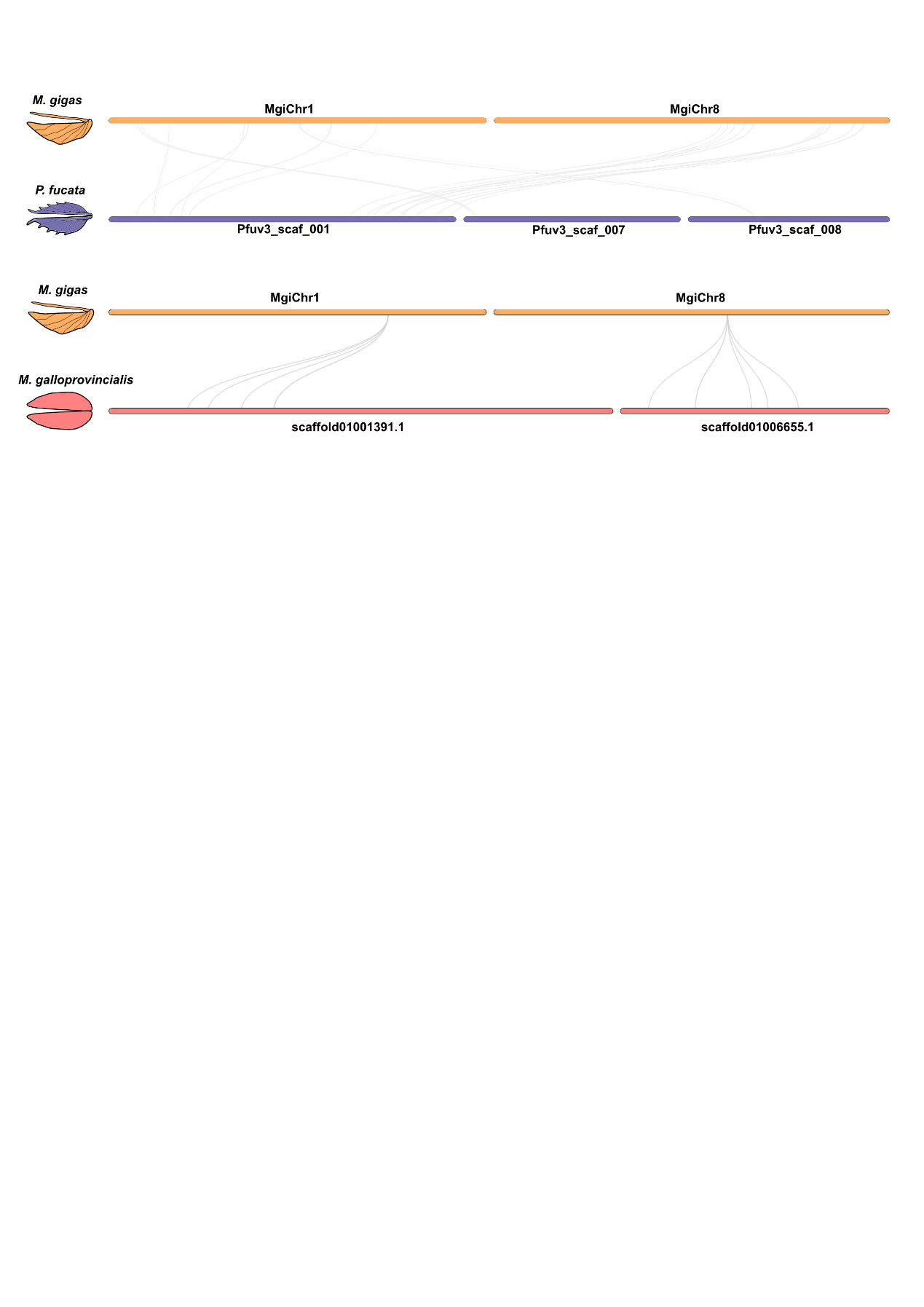
**

**Collinearity analysis of chromosomes that contain the target coding genes retrieved from *M. gigas*, *P. fucata* and *M. galloprovincialis*.** Grey lines interconnecting chromosomes represent gene blocks with a minimum of five orthologue genes. No homologue regions containing the target coding genes were found in the genomes of *P. fucata* and *M. galloprovincialis* (slightly asymmetrical and a symmetrical bivalve, respectively).

**Supplementary Figure 20**

**
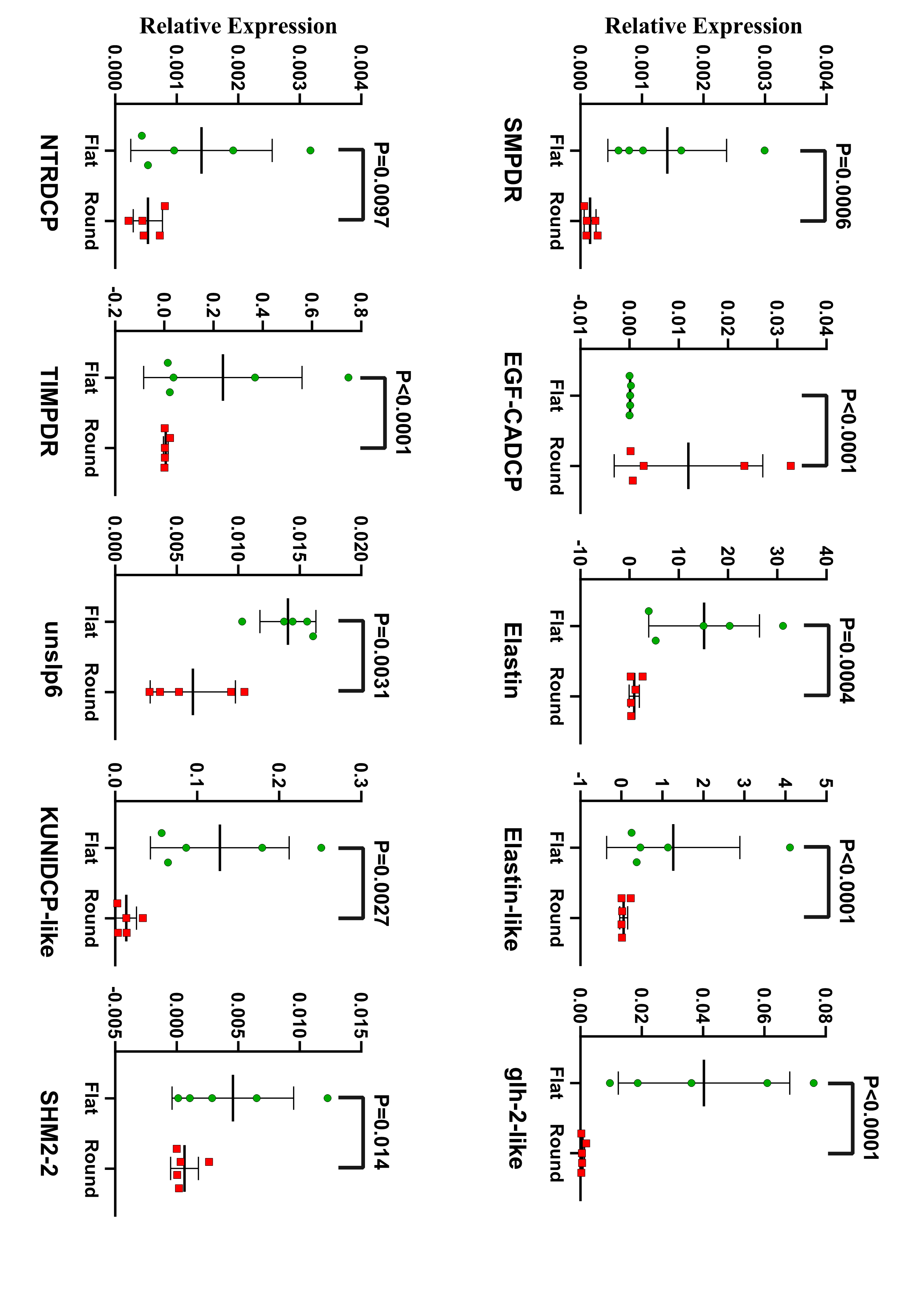
**

**Corroboration between expression levels of differentially expressed candidate genes in the mantle transcriptomes and by qRT-PCR analysis.** Expression of the ten candidate genes selected (including the candidate non-coding and protein coding transcripts) in the mantle from the flat (green) and round (red) valves by qRT-PCR. Each graph represents the expression profile of each gene in the mantle of the two shell sides. Relative gene expression was calculated as fold change after normalization with the average expression of the reference genes (EF1α and RL7), that were not significantly different across the samples. Statistical analysis was performed using a student t-test and the presented data corresponds to the mean ± SEM of n = 5 biological replicate.

**Supplementary Figure 21**

**
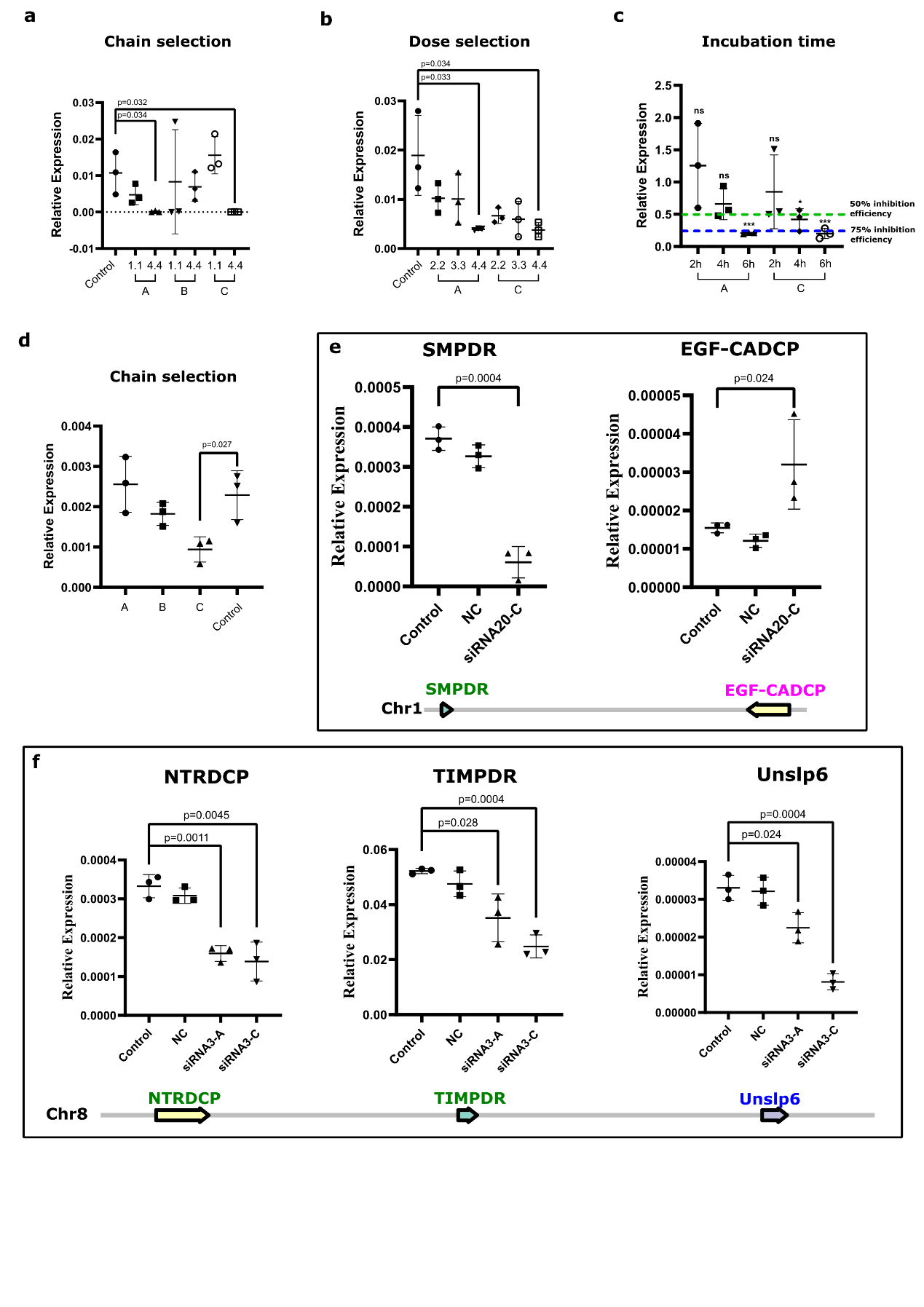
**

**Optimization of siRNA experiments (chain, dose, and incubation time) and effect of lncRNA ablation on candidate neighbour gene expression in *ex-vivo* mantle cultures. (a-c)** Determination of the optimal siRNA experimental conditions for *TIMPDR* based on its effect on the expression of *TIMPDR* transcripts in mantle *ex-vivo* tissue culture. Selection of (**a)** chains, (**b)** dose and (**c)** incubation time. **(a)** the effect on *TIMPDR* transcript abundance of the different siRNA nucleotide chains A, B and C (11mg/ml and 4.4 mg/ml) and (**b)** the effect of *TIMPDR* chain concentration (2.2 mg/ml, 3.3 mg/ml and 4.4 mg/ml) after 8 h incubation with mantle *ex-vivo* tissue culture. The expression data was determined by qRT-PCR and calculated as the fold change relative to the average of the expression of two reference genes (EF1α and RL7). The data is represented as the mean ± SEM of 3 biological replicates (each biological replicate represents a mixture of ± 20 mantle fragments from 6 individuals) and a student t-test was performed to determine the statistical difference between the lnc-treated samples compared to the control. (**c)** Effect of incubation time (2, 4 and 6h) on the effectiveness of the candidate chains to ablate *TIMPDR* expression. Expression data was calculated as the fold change relative to the control. Data is represented as the mean ± SEM of 3 biological replicates (each biological replicate represents a mixture of ± 20 mantle fragments from 6 individuals) and a student t-test was performed to determine the statistical difference between the samples compared to the control. (**d)** The effect of the three siRNA chains (A, B and C) for *SMPDR* on the gene expression of *SMPDR* transcripts in mantle tissue fragments. The optimal conditions (dose and incubation time) previously obtained for *TIMPDR* were assessed for use with *SMPDR*. (**e-f)** Effect of the optimized siRNAs experimental conditions on the gene expression level of targeted lncRNAs and candidate regulatory protein coding genes in mantle ex-vivo cultures for e) *SMPDR* and f) *TIMPDR*. The gene expression data was obtained using qRT-PCR and calculated as the fold change relative to the average of the expression of the reference genes (*EF1α* and *RL7*). Data is represented as the mean ± SEM of 3 biological replicates (each biological replicate represents a mixture of ± 20 mantle fragments from 6 individuals) and a student’s t-test was performed to determine if there were statistical differences between the lncRNA treated samples in relation to the control. The *p value* is shown, and statistical significance was considered when p < 0.05. The control group (seawater only) and the group incubated with the negative siRNA chain (NC) were used as negative controls.

**Reference**

1. B. Sui, *et al.*, A novel antiviral lncRNA, EDAL, shields a T309 O-GlcNAcylation site to promote EZH2 lysosomal degradation. *Genome Biol.* **21**, 1–36 (2020).

2. S. Andrews, FastQC: a quality control tool for high throughput sequence data (2010).

3. A. M. Bolger, M. Lohse, B. Usadel, Trimmomatic: a flexible trimmer for Illumina sequence data. *Bioinformatics* **30**, 2114–2120 (2014).

4. C. Peñaloza, *et al.*, A chromosome-level genome assembly for the Pacific oyster Crassostrea gigas. *Gigascience* **10**, giab020 (2021).

5. D. Kim, B. Langmead, S. L. Salzberg, HISAT: a fast spliced aligner with low memory requirements. *Nat. Methods* **12**, 357–360 (2015).

6. M. Pertea, *et al.*, StringTie enables improved reconstruction of a transcriptome from RNA-seq reads. *Nat. Biotechnol.* **33**, 290–295 (2015).

7. M. Friendly, Corrgrams: Exploratory displays for correlation matrices. *Am. Stat.* **56**, 316–324 (2002).

8. M. I. Love, W. Huber, S. Anders, Moderated estimation of fold change and dispersion for RNA-seq data with DESeq2. *Genome Biol.* **15**, 1–21 (2014).

9. H. Wickham, *ggplot2: elegant graphics for data analysis* (springer, 2016).

10. R. Kolde, M. R. Kolde, Package ‘pheatmap.’ *R Packag.* **1**, 790 (2015).

11. A. W. Skeffington, A. Donath, ProminTools: shedding light on proteins of unknown function in biomineralization with user friendly tools illustrated using mollusc shell matrix protein sequences. *PeerJ* **8**, e9852 (2020).

12. N. Merchant, *et al.*, The iPlant collaborative: cyberinfrastructure for enabling data to discovery for the life sciences. *PLoS Biol.* **14**, e1002342 (2016).

13. Y. Fang, M. J. Fullwood, Roles, Functions, and Mechanisms of Long Non-coding RNAs in Cancer. *Genomics, Proteomics Bioinforma.* (2016) https:/doi.org/10.1016/j.gpb.2015.09.006.

14. L. Li, *et al.*, Genome-wide discovery and characterization of maize long non-coding RNAs. *Genome Biol.* (2014) https:/doi.org/10.1186/gb-2014-15-2-r40.

15. A. Li, J. Zhang, Z. Zhou, PLEK: A tool for predicting long non-coding RNAs and messenger RNAs based on an improved k-mer scheme. *BMC Bioinformatics* (2014) https:/doi.org/10.1186/1471-2105-15-311.

16. Y. J. Kang, *et al.*, CPC2: A fast and accurate coding potential calculator based on sequence intrinsic features. *Nucleic Acids Res.* (2017) https:/doi.org/10.1093/nar/gkx428.

17. L. Sun, *et al.*, Utilizing sequence intrinsic composition to classify protein-coding and long non-coding transcripts. *Nucleic Acids Res.* (2013) https:/doi.org/10.1093/nar/gkt646.

18. D. M. Emms and S. Kelly. OrthoFinder: phylogenetic orthology inference for comparative genomics. Genome biology, 2019, 20: 1-14.

19. Z. Li, *et al.*, RNAi-mediated knock-down of the dopamine beta-hydroxylase gene changes growth of razor clams. *Comp. Biochem. Physiol. Part B Biochem. Mol. Biol.* **252**, 110534 (2021).

20. K. Yamaguchi, Shell structure and behaviour related to cementation in oysters. *Mar. Biol.* **118**, 89–100 (1994).
